# Different and unified responses of soil bacterial and fungal community composition and predicted functional potential to 3 years’ drought stress in a semiarid alpine grassland

**DOI:** 10.3389/fmicb.2023.1104944

**Published:** 2023-03-14

**Authors:** Qian Wan, Lei Li, Bo Liu, Zhihao Zhang, Yalan Liu, Mingyu Xie

**Affiliations:** ^1^State Key Laboratory of Desert and Oasis Ecology, Xinjiang Institute of Ecology and Geography, Chinese Academy of Sciences, Urumqi, China; ^2^Xinjiang Key Laboratory of Desert Plant Roots Ecology and Vegetation Restoration, Xinjiang Institute of Ecology and Geography, Chinese Academy of Sciences, Urumqi, China; ^3^Cele National Station of Observation and Research for Desert-Grassland Ecosystems, Cele, China; ^4^University of Chinese Academy of Sciences, Beijing, China; ^5^Shandong Provincial Key Laboratory of Soil Conservation and Environmental Protection, College of Resources and Environment, Linyi University, Linyi, China

**Keywords:** bacteria and fungi, drought stress, microbial community, diversity, functional potential, alpine grasslands

## Abstract

**Introduction:**

Soil microbial communities are key to functional processes in terrestrial ecosystems, and they serve as an important indicator of grasslands status. However, the responses of soil microbial communities and functional potential to drought stress in semiarid alpine grasslands remain unclear.

**Methods:**

Here, a field experiment was conducted under ambient precipitation as a control, −20% and −40% of precipitation to explore the responses of soil microbial diversity, community composition, and predicted functional potential to drought stress in a semiarid alpine grassland located in the northwest of China. Moreover, 16S rRNA gene and ITS sequencing were used to detect bacterial and fungal communities, and the PICRUST and FUNGuild databases were used to predict bacterial and fungal functional groups.

**Results:**

Results showed drought stress substantially changes the community diversity of bacteria and fungi, among which the bacteria community is more sensitive to drought stress than fungi, indicating that the diversity or structure of soil bacteria community could serve as an indicator of alpine grasslands status. However, the fungal community still has difficulty maintaining resistance under excessive drought stress. Our paper also highlighted that soil moisture content, plant diversity (Shannon Wiener, Pieiou, and Simpson), and soil organic matter are the main drivers affecting soil bacterial and fungal community composition and predicted functional potential. Notably, the soil microbial functional potential could be predictable through taxonomic community profiles.

**Conclusion:**

Our research provides insight for exploring the mechanisms of microbial community composition and functional response to climate change (longer drought) in a semiarid alpine grassland.

## 1. Introduction

Precipitation is the main water resource of arid and semiarid land, and it plays an important key driving factor for various biological processes at different spatial and temporal scales (Harpole et al., 2007). The intensification of human activities has substantially affected the global atmospheric circulation pattern (IPCC, 2013) and enhanced the variation of global precipitation (Easterling et al., 2019). Climate models have also predicted the variability in global precipitation increases with a considerable increase in the intensity, frequency, and duration of drought events in the future (Huang et al., 2016). Arid and semiarid land grassland ecosystem plays an important role in maintaining the stability of the ecosystem structure and service function (Prevey, 2019). Numerous previous studies have shown drought has a profound effect on plant diversity (Grman et al., 2010), nutrient cycling (Haverd et al., 2017), and microbial communities (Yuste et al., 2011) in arid and semiarid land ecosystems, which restricts its sustainable development.

Soil microorganisms play an important role in nutrient cycling and organic matter decomposition in soil–plant systems. Water is indispensable for the growth, metabolism, and reproduction of soil microorganisms, and precipitation changes directly affect soil microbial communities (Wardle et al., 2004). In general, evidence suggests bacterial diversity decreases under drought stress with resource constraints (Raúl et al., 2018). Soil fungal community shows higher stability compared with bacteria under drought conditions (Yuste et al., 2011). For example, a 3-year experiment showed precipitation variation has a strong effect on bacteria but not on fungi in a meadow grassland in northeastern China (Yang et al., 2021). Furthermore, fungal community diversity increases because of high drought tolerance, particularly in extreme arid environments (Preece et al., 2019). However, some studies have shown fungal and bacterial communities are resistant to drought stress (Abbasi, 2020). Drought does not affect the community diversity of bacteria and fungi in semiarid temperate grassland ecosystems (Li et al., 2022a). Based on existing research, the response of soil microbial community diversity to drought stress remains unclear. Therefore, understanding the mechanism how drought affects soil microbial community diversity is essential for predicting the effects of climate change in alpine grassland ecosystems.

Soil microorganisms adapt or resist external drought stress by changing their community composition. The observed changes in soil microbial community composition involve variations in the relative abundance of the dominant phyla, which could be a consequence of drought stress. For example, drought stress increases the relative abundance of the Ascomycota phylum fungal community in a semiarid grassland (Chen et al., 2019). The relative abundance of Actinobacteria, as a dominant phylum in arid soils, decreased substantially with more mean annual precipitation in the grassland of the Loess Plateau (Li et al., 2020). Soil microbial communities are highly variable in natural and experimental environments because of the different duration of drought stress. More tolerant soil microbial bacterial and fungal phyla (i.e., Actinobacteria, Glomeromycota, and Ascomycota) are selected under long-term periodic drought to achieve a resistance memory to drought (Canarini et al., 2021). Therefore, understanding the responses of soil microbial communities to various degrees of drought stress in sensitive alpine grasslands is lacking.

Drought-induced changes in microbial communities may be regulated through two pathways. Firstly, soil properties such as soil moisture (Naidoo et al., 2022), pH (Wang et al., 2015) and temperature (Zhang et al., 2013) are major drivers of microbial communities. In addition, drought may affect soil microbial communities by regulating plant characteristics, such as plant coverage (Maestre et al., 2016), plant diversity (Spehn et al., 2000), and plant biomass (Na et al., 2019). The diversity and abundance of soil bacteria and fungi are decreased under drought stress because of reduced plant cover and soil organic carbon input (Maestre et al., 2016). The variation in plant diversity affects plant products and organic components, thereby influencing soil microbial composition (Spehn et al., 2000). However, studies have indicated that soil microbial composition is not substantially related to plant species diversity (Meier and Bowman, 2008) but significantly correlated with multi-species litter mixtures (Mathieu et al., 2017). Therefore, considering the soil–plant–microbial relationship, the regulating characteristics of abiotic factors (i.e., soil properties) and biotic factors (i.e., plant characteristics) to soil microbial community in alpine grassland under drought stress must be comprehensively explored.

Microbial functional potential is mainly affected by soil microbial community structure and composition. A research stated that taxonomy and function are coupled (Fierer et al., 2012), indicating that microbial functional potential changes can be directly predicted by monitoring changes in microbial community classification. A study showed that changes in precipitation patterns can affect the microbial community composition and functional potential (β-diversity) in desert soils; for example, Acidobacteriota and “resistance to antibiotics and toxic compounds” related genes are relatively more abundant under an increased precipitation zone (Naidoo et al., 2022). Another research determined that microbial functional (at the β-diversity level) is strongly correlated with taxonomic and phylogenetic β-diversity in many soils, including cold deserts, hot deserts, forests, grasslands, and tundra (Fierer et al., 2012). However, the relationship between soil microbial community composition and functional potential under drought stress in arid and semiarid regions is still unclear. Thus, the responses of soil microbial community composition and functional potential under drought stress must be explored, and the coupled mechanism must be clarified.

A 3-year field *in situ* control experiment was conducted to select three precipitation gradients (100% referred to ambient precipitation, −40% and −20%) of drought treatment in the northern slope of Kunlun Mountains. Moreover, 16S rRNA gene and ITS sequencing were used to detect bacterial and fungal communities, and the PICRUST and FUNGuild databases were used to predict bacterial and fungal functional groups. Here, we predict that (1) drought stress will change soil bacterial and fungal community diversity and composition, and bacterial community is more sensitive to drought compared with fungal community structure; (2) biotic and abiotic factors together influence soil microbial community; and (3) there is a coupling between soil microbial community structure and microbial functional potential.

## 2. Materials and methods

### 2.1. Study area description and experimental design

This paper is based on the rainfall experiment platform of the national grassland fixed monitoring station of Xinjiang Institute of Ecology and Geography, Chinese Academy of Sciences. The experimental site is located on the northern slope of the Kunlun Mountains (80°35′08″ E, 36°08′02″ N) at an altitude of 3,236 m, which is influenced by a typical continental arid climate. The mean annual precipitation is approximately 335 mm, which occurs during the growing season (April to September, Supplementary Table S1). The soil is a moderately mature gray desert soil, and 0–20 cm of the soil is sandy loam (Wan et al., 2022). The predominant vegetation types are *Seriphidium rhodanthum*, *Stipa capillata*, *Astragalus polycladus,* and *Allium chrysanthum* Regel.

The three treatments in the rainfall platform as described by Zhang et al. (2019) and modified for this study included a control (CK, natural precipitation), −20% (D20, 20% reduction of precipitation), and −40% (D40, 40% reduction of precipitation) with each experimental treatment replicated four times. Supplementary Figure S1 shows a rainfall platform with 2 m × 3 m size was randomly assigned in the study area with a 6 m buffer established between each neighboring plots, ensuring excess precipitation from the rainfall platform dropping into the buffer. The rain shelters were installed at 1.2 m above ground, and 20% and 40% of the plot area were covered by a transparent tempered glass (Supplementary Figure S1), minimizing light blockage and avoiding temperature increase. The whole experimental area was flat, and the natural slope was less than 1%.

Temperature and humidity sensors were installed in each sample plot at a 15 cm soil depth to enable the real-time collection of soil moisture and temperature conditions (Supplementary Figure S2). The experimental treatments were conducted over 3 years starting in April 2019 and ending in September 2021 for the artificially controlled rainfall alteration experiment. To avoid differences in vegetation composition and soil properties caused by spatial heterogeneity, uniform grassland was fenced and divided into three blocks before the experiment. Moreover, four plots similar in vegetation composition were established in each block.

### 2.2. Plant and soil sample collection

In September 2021, as the end of the drought treatment, 1 m × 1 m subplots were randomly selected within the treatment plots, and the indicators of the vegetation survey included the composition and density of each species (Supplementary Table S2). In the vegetation survey, all the surviving aboveground plant individuals in each subplot were collected and dried at 65°C until constant weight to obtain aboveground biomass (AGB). Five soil samples were collected randomly at 0–20 cm by using a soil corer (2.1 cm inner diameter) in each plot and mixed into one composite sample. Four duplicate soil samples were collected of each treatment, producing 12 soil samples. Living root samples obtained from randomly selected soil plots (20 cm × 20 cm × 20 cm) were cleaned with deionized water and dried to a constant weight at 65°C to obtain belowground biomass (BGB). Soil samples passed through a 2 mm sieve were equally divided into two groups. The first group, which was dried in air at room temperature, was used to determine the soil total nitrogen (TN) and total phosphorus (TP), and the second fresh soil group was stored at −80°C for soil microbial community.

### 2.3. Soil physicochemical characteristics

Soil moisture was measured by the drying method (105°C for 48 h). Soli organic matter (SOM) and TP were determined by potassium dichromate heating and acid digestion (Kalembasa and Jenkinson, 1973), respectively. TN was determined by using an automatic elemental analyzer (Vario EL Cube, Elementar, Langenselbold, Germany). Total potassium (TK) and pH (PHS–3C; Shanghai) were determined by flame photometry and electrode potentiometry (Walker and Adams, 1958), respectively. Soil available phosphorus (SAP) was leached with 0.5 mol L^−1^ of NaHCO_3_ (pH = 8.5) and determined by molybdenum blue colorimetry (Kjeldahl, 1883). Twelve samples including four replicates per treatment were analyzed. All changes in soil and plant properties under different stages of precipitation were collected as shown in Supplementary Table S3.

### 2.4. Soil DNA extraction and bacterial community composition analysis

Total soil DNA was extracted using the TIANamp Soil DNA Kit (TIANGEN) according to the manufacturer’s protocol, and each treatment included four samples with each sample extracted once. Concentration quality and DNA purity were evaluated using a NanoDrop One spectrophotometer (Thermo Scientific, Wilmington, DE, United States) and through agarose 1% gel electrophoresis (180 V, 25 min). Bacterial 16S rRNA gene and fungal ITS sequences were used for PCR amplification using different primers. For bacterial diversity analysis, the primer sets 338F and 806R (Supplementary Table S4) were used to amplify 16S rRNA gene (Canarini et al., 2021). The fungal sequences of the ITS-V1 gene (Yuste et al., 2011) were amplified using the universal primers ITS5 and ITS2 (Supplementary Table S4). The PCR products for each sample were mixed after completing PCR amplifications using the same template with three replicates and then purified using the Thermo Scientific GeneJET PCR Purification Kit (Li et al., 2014). High-throughput sequencing analysis of the target genes was applied using the Illumina NovaSeq PE250 platform (Shanghai Personalbio Technology Co., Ltd.) with the paired-end 300 bp strategy (Reyon et al., 2012). Bioinformatic analysis was performed using QIIME2 (2019.4). The raw data were obtained after sequencing. Firstly, the primer fragments were cut, and the mismatched primer sequences were discarded through the function of qiime cut-adaptive trim-pair. Then, the Divisive amplicon Denoise Algorithm 2 (DADA2) was used to perform sequence quality control, denoising, splicing, and chimera removal through the qiime dada2 denoise-pair function (Callahan et al., 2016). DADA2 no longer clustered in similarity, and only dereplication or clustering in 100% similarity was performed (Callahan et al., 2016). Based on QIIME2 (2019.4), Vsearch (v2.13.4 linux_x86_64) and cutadapt (v2.3) were used for subsequent analysis, which clustered high-quality sequences at 97% similarity level and output representative sequences and amplicon sequence variant (AVS) tables (Edgar, 2017). Singletons were removed from AVS tables and their representative sequences for downstream analysis. Bacteria and fungi were performed using QIIME 2’s classify-sklearn algorithm (Bokulich et al., 2018) based on Greengenes and UNITE databases, respectively, and unleveled ASV sequence was selected for species annotation in QIIME2 software through the pretrained Naive Bayes classifier. The raw data were submitted to the National Center for Biotechnology Information (NCBI).^1^

### 2.5. Statistical analysis

Single-factor analysis of variance was performed using Duncan’s multiple range test (*p* < 0.05) in SPSS 26.0 (SPSS Inc., Chicago, IL, United States). The alpha diversity of the microbial community was estimated with Shannon index based on the Bray–Curtis distance.^2^ Nonmetric multidimensional scaling (NMDS) analysis was performed in accordance with the Bray–Curtis distance matrix to visualize the microbial communities, and the differences in microbial community composition were presented by performing an ordination plot using “ggplot2.” The significance of the separation between stages of microbial community structure was tested by the “ADONIS” function of the vegan R software package (999 permutations). Mantel test was used to investigate the relationship between the Shannon index of bacteria and fungi with environmental factors based on 9,999 permutations using the vegan R software package. Redundancy analysis (RDA) was used to assess the relationship between environmental factors and bacterial and fungal community structure using the vegan package (Oksanen et al., 2014). The significance of RDA correlations was tested Monte Carlo permutation test. Spearman’s correlation coefficient was used to test the relationship between environmental factors (plant and soil properties) with relative abundance of the top 10 bacteria/fungi at the phylum level. All data were processed using QIIME2 (2019.4) and Excel (2019), and plots were performed using Origin (Origin Laboratories, Ltd., Northampton City, MA, United States) and R (version 3.6.1). Bacterial function prediction was analyzed using PICRSt software and the closed AVS tables obtained by QIIME were compared with the KEGG database to obtain different database function prediction information (Langille et al., 2013). FUNGuild (Fungi Functional Guild) V1.0 online platform was used to classify fungi ecologically and functionally. OTUs obtained from high-throughput sequencing were uploaded to the FUNGuild platform for analysis, and the results were downloaded for screening fungal communities and linking fungal species classification to functional guild classification by bioinformatics methods (Sun et al., 2016). Heat maps and histograms were plotted by using the heatmap package in R. Microbial network analysis was performed by the genes cloud tools.^3^ Only the top 100 abundance at the genus level were selected, and the co-occurrence patterns were explored based on strong (Spearman’s ρ > |0.6|) and significant correlations (*p* < 0.05). Cytoscape 3.4.0^4^ was used to visualize network.

## 3. Results

### 3.1. Soil microbial diversity under drought stress

The Illumina NovaSeq PE250 platform was used to filter the obtained raw data, obtaining 1,132,993 bacterial and 1,395,635 fungal high-quality sequences with averages of 94,416 and 116,302 sequences, respectively. The average coverage of all samples was more than 97%, and the rarefaction curve of each sample was flat (Supplementary Figure S3), indicating that the sequencing depth was saturated and could reflect the vast majority of microbial diversity information in the samples (Edgar, 2017). Drought stress (D20 and D40) significantly reduced bacterial species richness (*p* < 0.05), and the D20 treatment showed the lowest fungal species richness (*p* < 0.05, Supplementary Figure S4).

**Figure 1:**
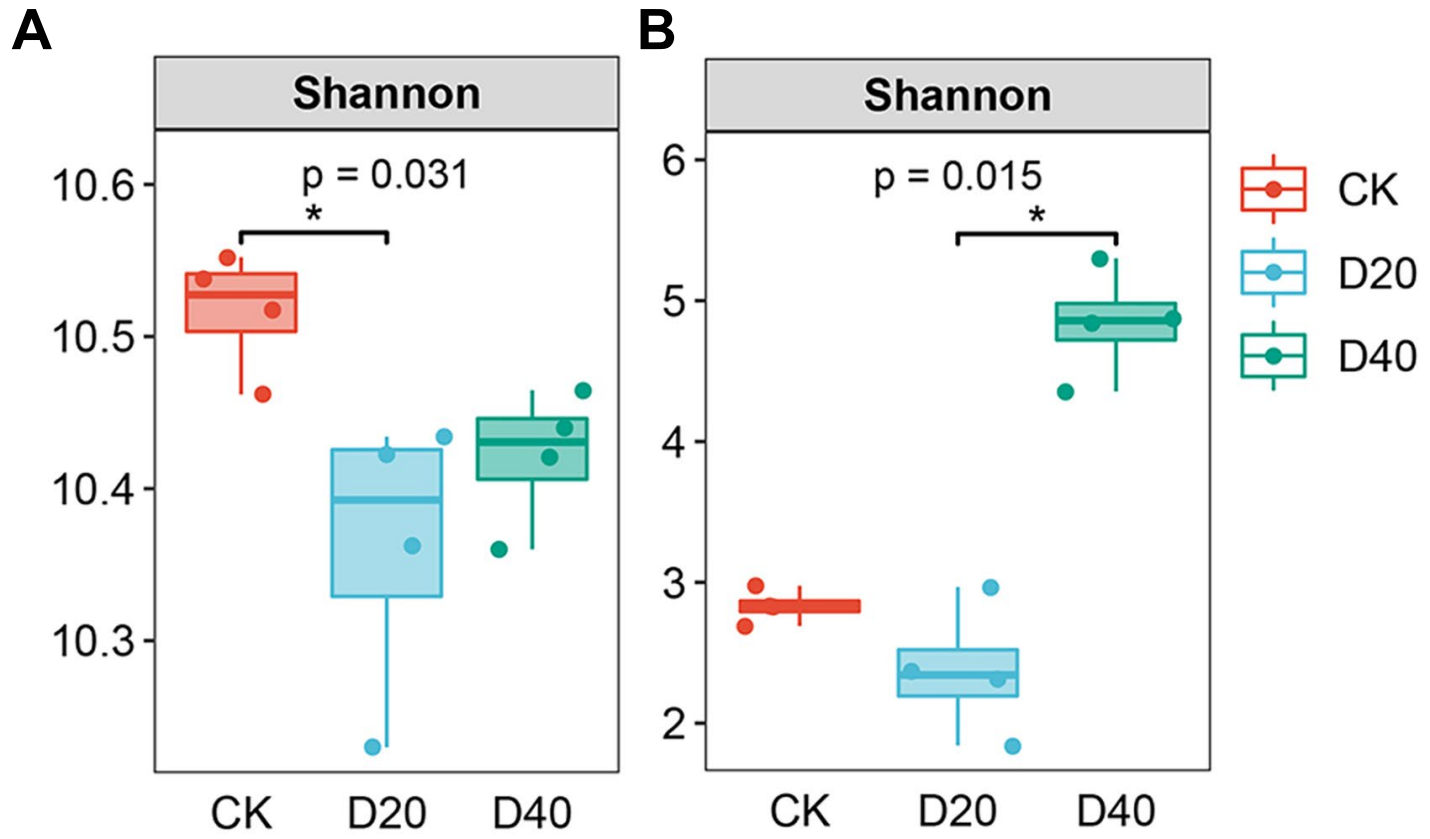
Alpha diversity index of soil microorganisms based on AVS. Changes in alpha diversity of bacteria and fungi in different stages of drought gradient. Shannon index of bacteria **(A)** and fungi **(B)**, respectively. CK: natural precipitation, D20: 20% precipitation reduction, and D40: 40% precipitation reduction.

The Shannon diversity index of bacteria and fungi in different treatments showed similar trends with species richness (Supplementary Figure S4). The Shannon diversity index for soil bacteria was highest in CK and lowest in D20 (*p* < 0.05, Figure 1A), indicating drought stress significantly reduced bacterial community diversity. Moreover, the Shannon diversity index for fungi was highest in D40 and lowest in D20 (*p* < 0.05, Figure 1B). Fungi were more resistant to drought compared with bacteria, especially in D20, but excessive drought (D40) may lead to the increasing of specific taxa and reorganization of fungal communities.

### 3.2. Soil microbial community structure under drought stress

**Figure 2:**
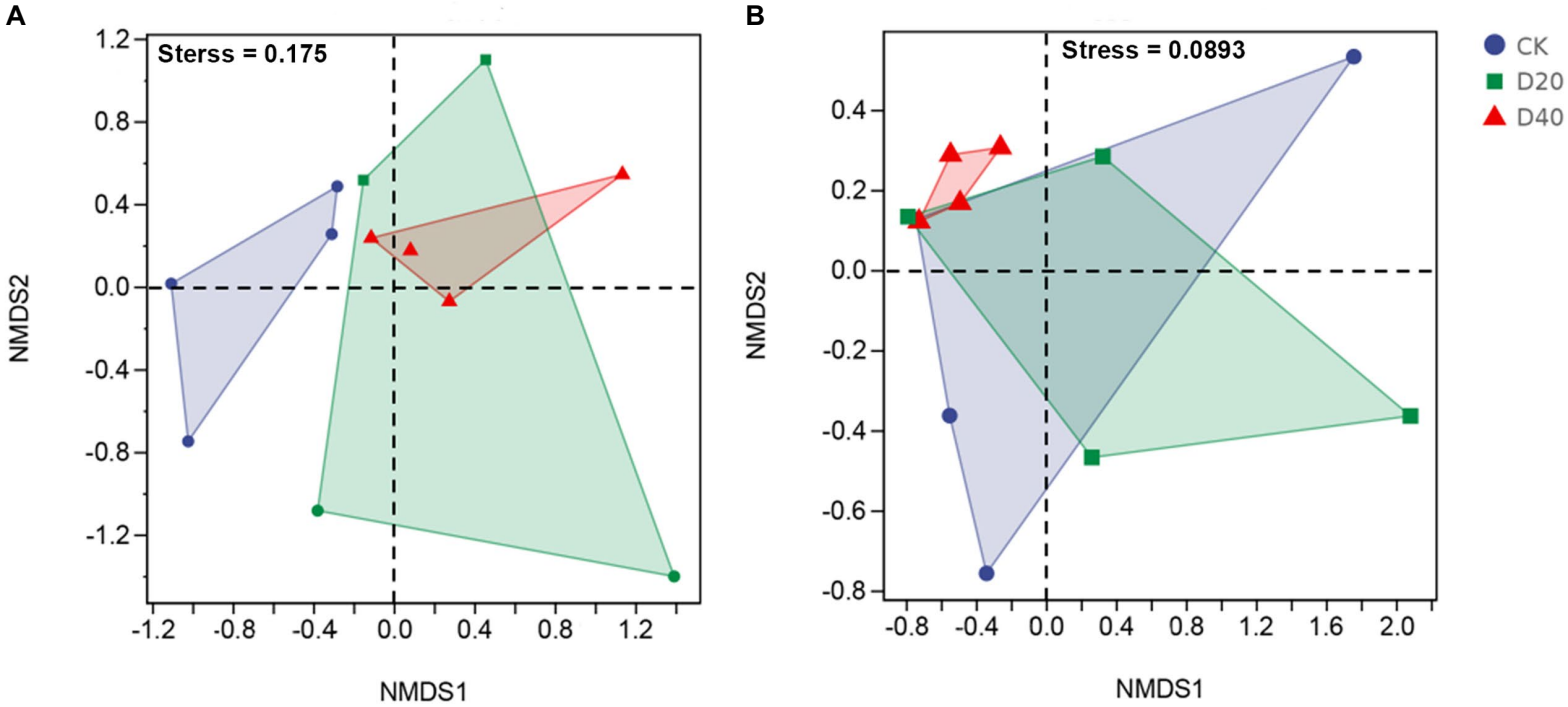
The non-metric multidimensional scaling (NMDS) analysis of bacterial and fungal communities at different stages of degradation. **(A)** bacterial community structure, **(B)** fungal community structure.

The NMDS results for bacterial community and fungi community showed a stress value of 0.175 (Figure 2A) and 0.089 (Figure 2B), which are suitable for NMDS analysis. In addition, nonparametric multivariate statistical tests (Adonis) indicated that bacterial (*p* = 0.086) and fungal (*p* = 0.106) community structures have no significant differences.

**Figure 3:**
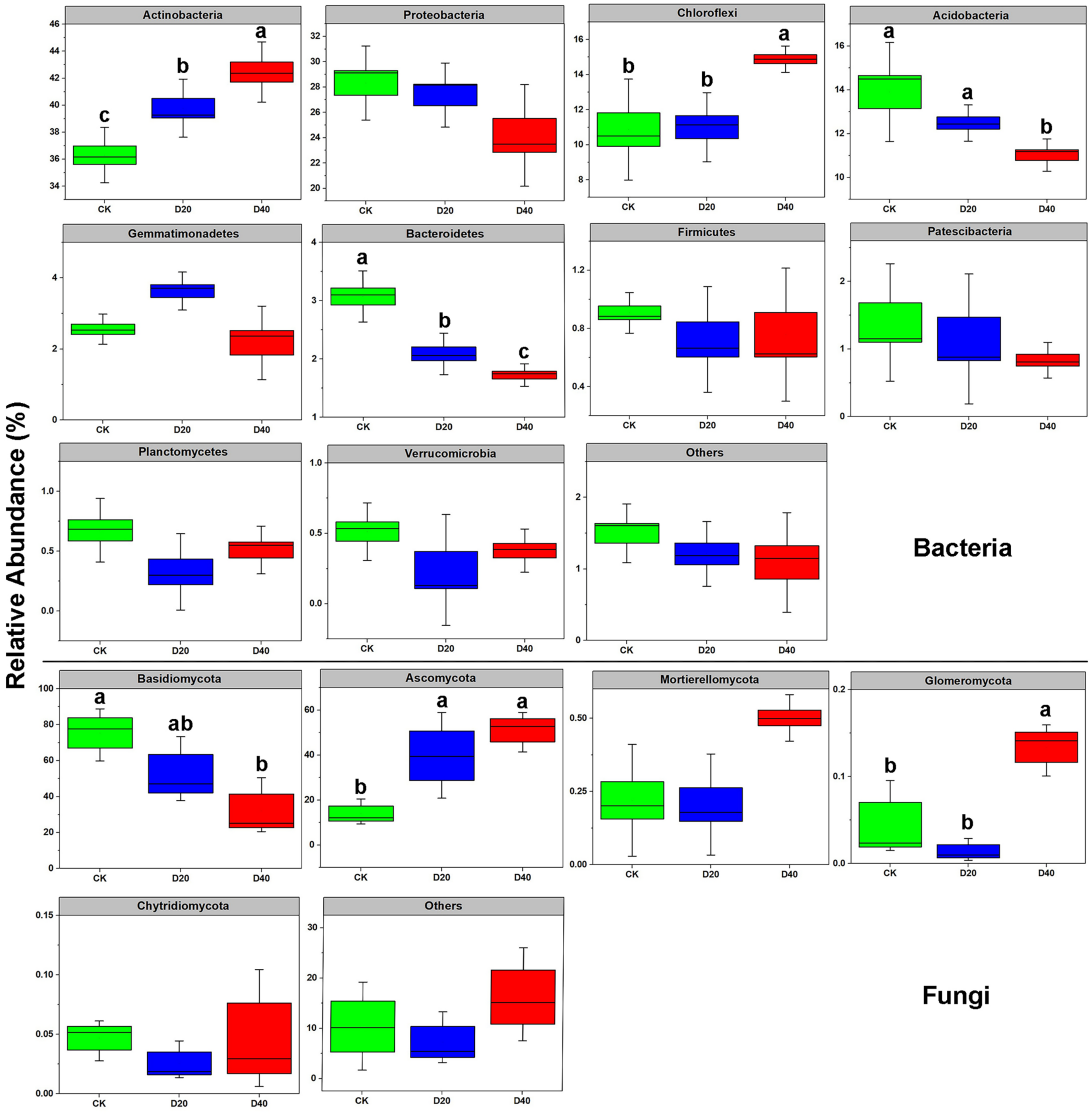
Relative abundance of bacterial and fungal phyla at different drought levels. Different lowercase letters (a, b, ab, c) indicate significant differences among the three drought levels (*p* < 0.05).

Further refinement of community structure showed the dominant bacterial phyla in soil for all treatments were Actinobacteria (36.30%–42.45%), Proteobacteria (24.17%–28.31%), Chloroflexi (10.86%–14.88%), and Acidobacteria (11.02%–13.90%), followed by the variable occurrence of Gemmatimonadetes, Bacteroidetes, Firmicutes, Patescibacteria, Planctomycetes, and Verrucomicrobia. Drought stress increased the relative abundance of Actinobacteria and Chloroflexi (*p* < 0.05) but reduced the relative abundance of Acidobacteria and Bacteroidetes (*p* < 0.05, Figure 3). At the genus level, only three of the top 10 bacterial species were influenced by drought stress. Subgroup-6 and A4 both declined, while Sphingomonas increased with drought gradient (*p* < 0.05, Supplementary Figure S5). The predominant phyla of fungi included Basidiomycota (30.56%–75.38%) and Ascomycota (13.97%–51.03%), followed by the variable occurrence of Mortierellomycota, Glomeromycota, and Chytridiomycota. In the fungal community, drought stress increased the relative abundance of Ascomycota and Glomeromycota (*p* < 0.05) but reduced the relative abundance of Basidiomycota (*p* < 0.05, Figure 3). At the genus level, only two of the top 10 fungi species were influenced by drought stress, showing as Hygrocybe declined with drought gradient, and Gibberella was lowest in D20 (*p* < 0.05, Supplementary Figure S5).

### 3.3. Environmental factors associated with soil microbial diversity and community structure

**Figure 4:**
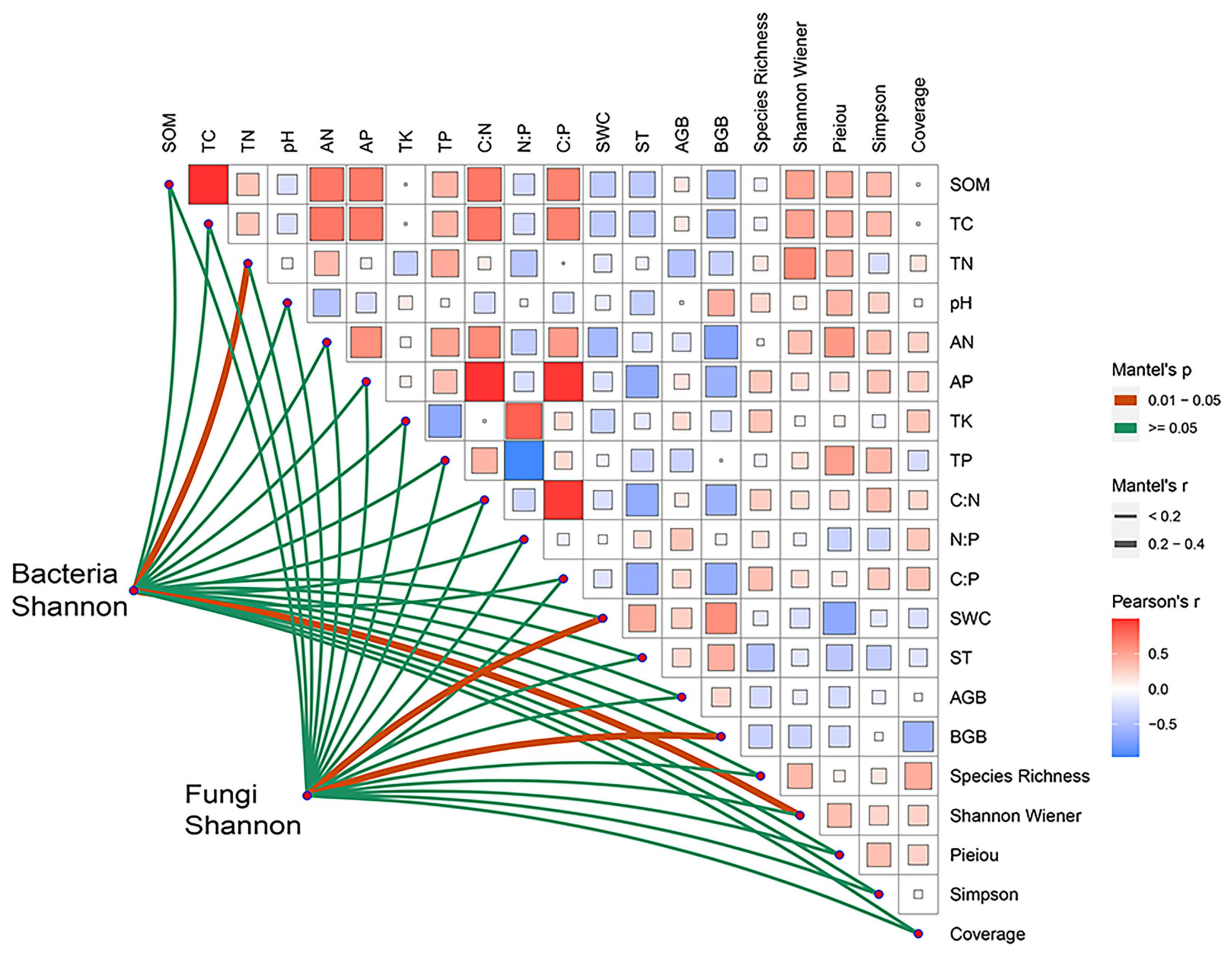
Relationship between the Shannon index of bacteria and fungi with environmental factors. SOM, soil organic matter; AN, soil alkali–hydrolysable nitrogen; AP, available phosphorus; TK, total soil potassium; TC, total soil carbon; TN, total soil nitrogen; TP, total soil phosphorus; soil total C: total N, soil total C: total P and soil total N: total P; SWC, soil moisture content; ST, soil temperature; AGB, aboveground biomass; BGB, belowground biomass.

In soil properties, soil bacterial Shannon index was significantly correlated with TN (*p* < 0.05), and fungal Shannon index was significantly correlated with SWC (*p* < 0.05, Figure 4). In addition, for plant factors, the Shannon indexes of the soil bacterial and fungal communities were significantly correlated with Shannon Wiener and BGB, respectively (*p* < 0.05, Figure 4). Furthermore, under drought treatments, no same environmental factor was correlated with the soil bacterial and fungal communities, indicating that the internal mechanisms of changes in soil microbial diversity and community structure under drought stress are different.

**Figure 5:**
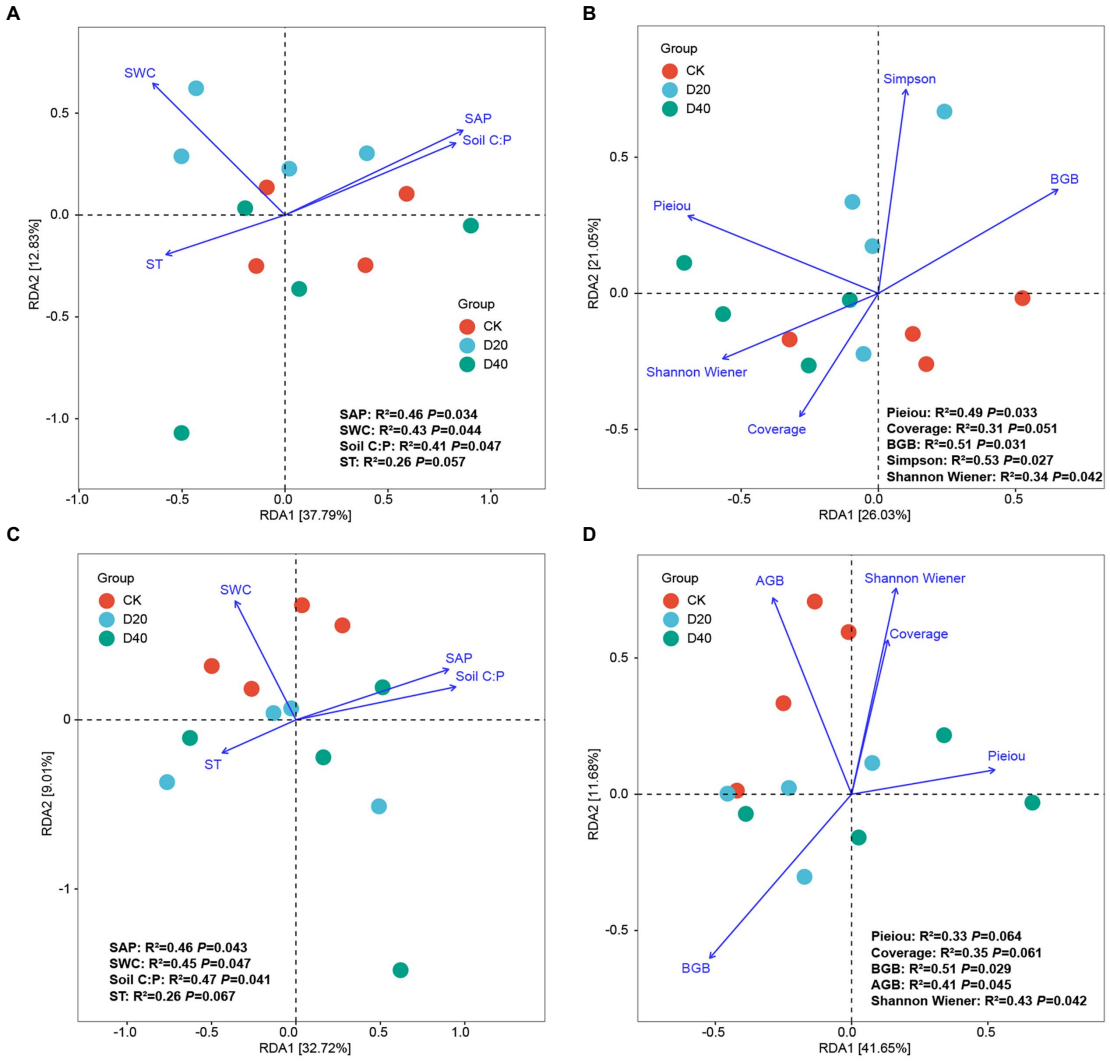
Ordination plots of RDA between microbiology communities, soil and plant properties at the AVS. **(A)** Bacterial community and soil factors, **(B)** Bacterial community and plant characteristics, **(C)** Fungal community and soil factors and **(D)** Fungi community and plant characteristics. SAP, available phosphorus.

RDA was applied to analyze the relationship between environmental factors and microbial community composition under drought stress. The RDA results explained 50.62% (37.79% for axis 1 and 12.83% for axis 2) and 47.08% (26.03% for axis 1 and 21.05% for axis 2) of the relationship between soil bacterial community composition with soil factors and plant factors (Figures 5A,B). In the soil bacterial community, soil C:P (R^2^ = 0.41, *p* = 0.047), SAP (R^2^ = 0.46, *p* = 0.034), and SWC (R^2^ = 0.43, *p* = 0.044) are the main soil factors affecting the bacterial community structure. Moreover, plant diversity [Simpson (R^2^ = 0.53, *p* = 0.027), Shannon Wiener (R^2^ = 0.34, *p* = 0.042), and Pieiou (R^2^ = 0.49, *p* = 0.033)] and BGB (R^2^ = 0.51, *p* = 0.031) are important plant factors controlling the soil bacterial community structure. However, under different drought stress, the positive correlation between SWC and D20 was significantly greater than that with CK and D40 (Figure 5A). In addition, D40 showed a higher positive correlation with plant factors (Pieiou, Shannon Wiener, and Coverage) than CK and D20 (Figure 5B). For the fungal community, the RDA results explained 41.73% (32.72% for axis 1 and 9.01% for axis 2) and 53.33% (41.65% for axis 1 and 11.68% for axis 2) of the relationship between soil fungal community diversity with soil factors and plant factors (Figures 5C,D). In the soil fungal community, SWC (R^2^ = 0.45, *p* = 0.047), soil C:P (R^2^ = 0.47, *p* = 0.041), and SAP (R^2^ = 0.46, *p* = 0.043) are the main soil factors affecting the fungal community structure. Shannon Wiener (R^2^ = 0.43, *p* = 0.042), BGB (R^2^ = 0.51, *p* = 0.029), and AGB (R^2^ = 0.41, *p* = 0.045) are important plant factors controlling the soil fungal community structure. Moreover, under different drought stress, the positive correlation between SWC and CK was the highest (Figure 5C), and the positive correlation between CK and plant factors (AGB and Shannon Wiener) was higher than that between D20 and D40 (Figure 5D). Thus, among all environmental attributes, especially under drought treatment, SWC and plant diversity are important determinant of the bacterial and fungal community structure.

**Figure 6:**
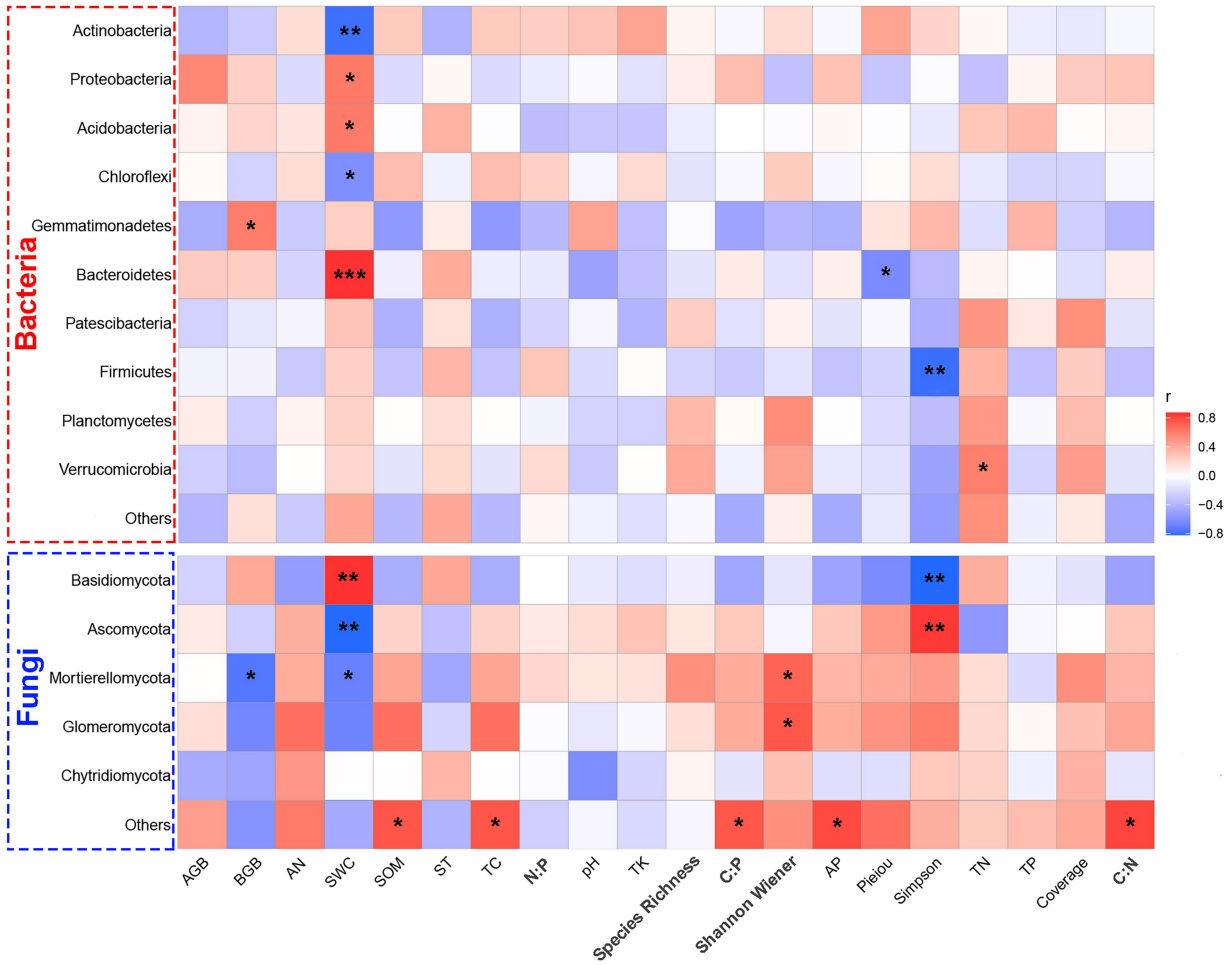
Correlations of plant and soil properties with relative abundance of the top 10 bacteria/fungi at the phylum level. The right side of the legend is the color range of R-values. SOM, soil organic matter; AN, soil alkali-hydrolysable nitrogen; AP, available phosphorous; TK, total soil potassium; TC, total soil carbon; soil total C: total N, soil total C: total P and soil total N: total P; ST, soil temperature; SWC: soil water content; SOC: soil organic carbon; TN: total nitrogen; TP: total phosphorus; AGB, aboveground biomass; BGB, belowground biomass. Significant correlations are reported as: *, *p* < 0.05; **, *p* < 0.01; and ***, *p* < 0.001.

Further refinement of the Spearman’s correlation between bacterial and fungal phylum with environmental factors showed the relative abundances of drought-tolerant bacterial phyla; for example, Acidobacteria (*r* = −0.7973, *p* = 0.0018) and Chloroflexi (*r* = −0.6116, *p* = 0.0345) showed a significant negative correlation with SWC. The relative abundances of drought-sensitive bacterial phyla, for example, Proteobacteria (*r* = 0.6208, *p* = 0.0312) and Bacteroidetes (*r* = 0.8582, *p* = 0.0003), were positively correlated with SWC, but Bacteroidetes (*r* = −0.6343, *p* = 0.0003) also showed a negative correlation with plant Pieiou. Moreover, the relative abundance of Firmicutes showed a significant negative correlation with plant Simpson richness (*r* = −0.8054, *p* = 0.0015; Figure 6). About fungal community composition, the abundance of Basidiomycota was positively correlated with SWC (*r* = 0.7797, *p* = 0.0027) in soil factors but negatively correlated with Simpson richness (*r* = −0.5385, *p* = 0.0071). However, the abundance of Ascomycota was positive correlation with Simpson richness (*r* = 0.6448, *p* = 0.0023) and negatively correlated with SWC (*r* = −0.7313, *p* = 0.0068). Furthermore, the abundance of Mortierellomycota was significantly and negatively correlated with BGB (*r* = −0.7161, *p* = 0.0088) of plants and SWC (*r* = −0.7509, *p* = 0.0048) but positively correlated with Shannon Wiener (*r* = 0.5059, *p* = 0.0439) of plants. Thus, the main environmental limiting factors (SWC and plant diversity) are consistent with the RDA results (Figure 5).

### 3.4. Soil microbial predicted functional potential under drought stress

**Figure 7:**
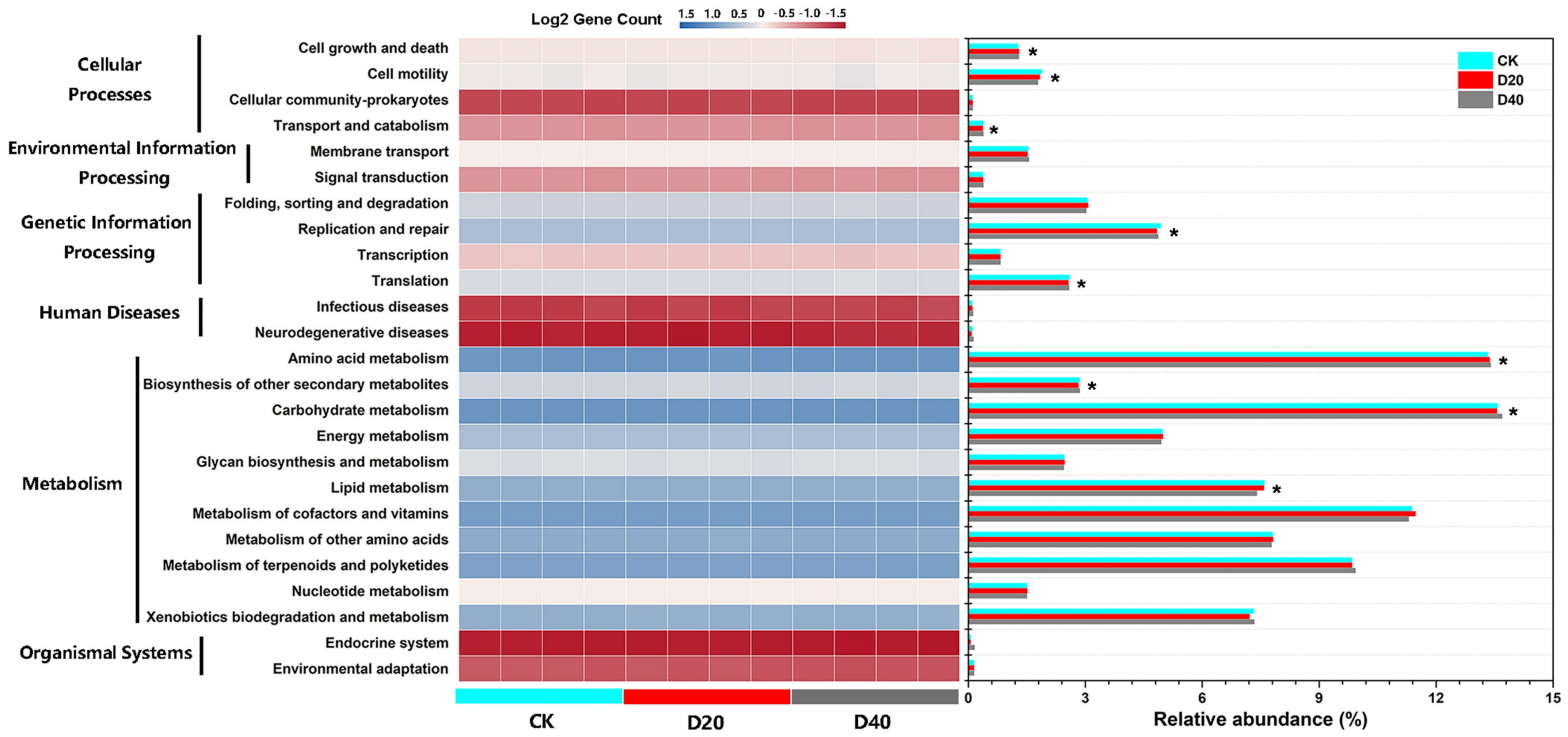
Relative abundance of predicted functional categories for different treatments in three origins bacterial microbiome using PICRUSt grouped into level-2 functional categories. * indicates the level-2 functional categories show a significant difference among the three drought levels.

PICRUSt was used to predict the bacterial community functions based on KEGG pathways genes, and six types of biological metabolic pathways were obtained: Metabolism, Genetic information processing, Environmental information processing, Cellular processes, Organismal systems, and Human diseases. Among them, the top two most abundant functions were Metabolism and Genetic information processing, accounting for 82.13%–83.08% and 11.23%–11.45%, respectively (Figure 7). The relative abundance in the secondary predicted functional layer was analyzed, and the heat map of all 25 predicted functions showed nine subfunctions with substantial differences under drought stress were mapped to three level-1 functional categories (Metabolism, Genetic information processing, and Cellular processes; Supplementary Table S5). Among them, the frequency of three predicted level-1 functional categories (Cell growth and death, Amino acid metabolism, and Carbohydrate metabolism) increased with drought gradient (*p* < 0.05, Supplementary Table S5). The frequency of three predicted level-1 functional categories (Cell motility, Replication and repair, and Lipid metabolism) declined with drought gradient (*p* < 0.05, Supplementary Table S5).

The functional classification of the fungi and the abundance of each functional classification in different treatments samples were obtained from the FUNGuild functional prediction (Table 1; Supplementary Figure S6). In the above functional classification, saprotroph and pathogen–saprotroph fungi were overwhelmingly represented in all treatments (Table 1; Supplementary Figure S6), consistent with the increased relative abundance of Ascomycota under drought stress (Figure 3). The fungal function under D40 treatment showed that the proportion of pathogen–saprotroph, endophyte–saprotroph, and parasite–saprotroph fungi increased, indicating that the fungal community still had difficulty maintaining resistance under excessive drought stress.

**Table 1:** Changes of predicted soil fungal functional groups in different treatments.

Fungal functional groups	CK (%)	D20 (%)	D40 (%)
Wood saprotroph	0.15 ± 0.01b	0.06 ± 0.01c	0.22 ± 0.01a
Saprotroph-undefined biotroph	71.76 ± 7.89b	81.70 ± 8.32a	43.09 ± 4.67c
Undefined saprotroph	10.11 ± 1.63b	6.14 ± 0.78c	27.51 ± 2.71a
Plant pathogen-wood saprotroph	7.12 ± 0.78b	9.11 ± 0.81a	2.01 ± 0.23c
Plant pathogen	3.22 ± 0.22a	0.33 ± 0.01b	3.37 ± 0.31a
Leaf saprotroph-plant pathogen	2.05 ± 0.23b	1.16 ± 0.14c	6.00 ± 0.89a
Fungal parasite-plant pathogen-plant saprotroph	1.40 ± 0.12a	0.08 ± 0.01c	0.76 ± 0.05b
Endophyte-litter saprotroph-soil saprotroph	0.28 ± 0.01b	0.22 ± 0.01c	0.81 ± 0.03a
Endophyte	0.02 ± 0.01b	0.01 ± 0.01b	0.24 ± 0.01a
Dung saprotroph-plant saprotroph	1.62 ± 0.15b	0.08 ± 0.01c	10.11 ± 1.26a
Animal pathogen-undefined saprotroph	0.25 ± 0.01a	0.03 ± 0.01c	0.07 ± 0.01b
Animal pathogen-plant saprotroph	0.90 ± 0.01b	0.62 ± 0.02c	2.72 ± 0.51a
Animal pathogen-plant pathogen-soil saprotroph	0.09 ± 0.01b	0.09 ± 0.01b	0.31 ± 0.01a
Animal pathogen-fungal parasite-undefined saprotroph	0.38 ± 0.01c	0.08 ± 0.01b	0.98 ± 0.08a

Different letters (a, b, c) indicate significant differences among different treatments (*p* < 0.05). Values are means ± SD (*n* = 4).

### 3.5. Environmental factors associated with soil microbial functional potential

**Figure 8:**
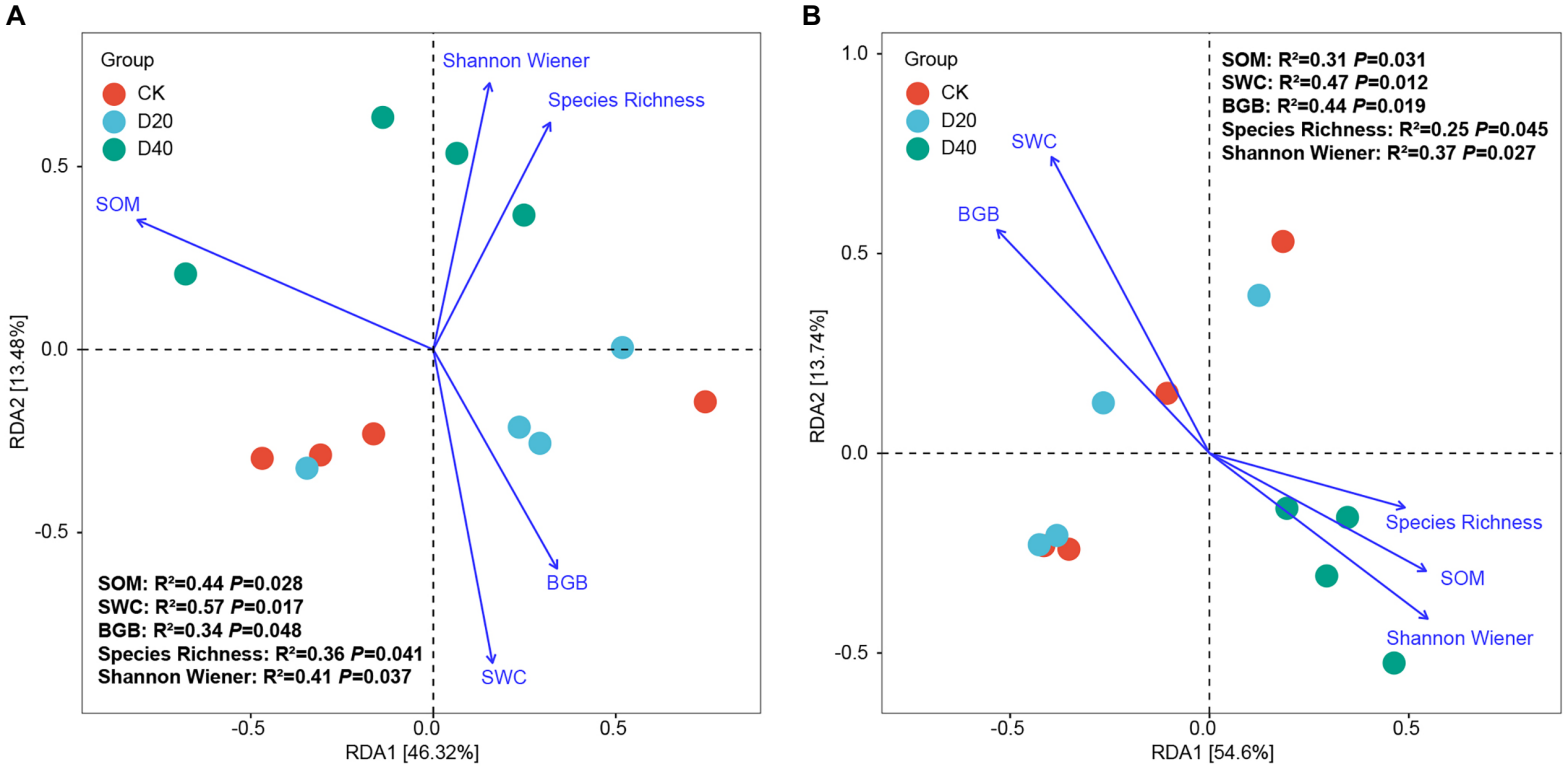
Ordination plots of RDA between predicted soil microbial functional potential and environmental factors at the AVS. **(A)** Bacterial functional potential and environmental factors, **(B)** Fungal functional potential and environmental factors.

To determine the major environmental factors associated with predicted soil microbial functional potential, RDA also was applied to analyze relationship between environmental factors, which significantly correlated with microbial community diversity and structure (Figure 5), with soil microbial functional potential. The RDA results explained 59.80% (46.32% for axis 1 and 13.48% for axis 2) and 68.34% (54.60% for axis 1 and 13.74% for axis 2) of the relationship between bacterial community functions (Figure 8A) and fungal community functions (Figure 8B) with environmental factors. The main important environmental factors controlling predicted bacterial and fungal functions were SWC, SOM, BGB, Species Richness, and Shannon Wiener. For the bacterial community functions, under different drought stress, compared with CK and D20, D40 showed higher correlation with SOM (R^2^ = 0.44, *p* = 0.028), Species Richness (R^2^ = 0.36, *p* = 0.041), and Shannon Wiener (R^2^ = 0.41, *p* = 0.037). For the fungal community functions, under different drought stress, D40 also showed higher correlation with SOM (R^2^ = 0.31, *p* = 0.031) and Shannon Wiener (R^2^ = 0.37, *p* = 0.027) than CK and D20. The functions of the bacterial and fungal communities under excessive drought stress (D40) both showed a correlation dependence on SOM and Shannon Wiener, which may be closely related to the death and reuse of plants and soil microorganisms.

## 4. Discussion

### 4.1. Microbial diversity of the dominant microbial phylum

The soil microbial diversity index is an important indicator for evaluating soil microbial community (Zhang et al., 2016). Previous studies have shown bacterial community diversity is more susceptible to drought compared with fungi (Kaisermann et al., 2017). Our results showed drought stress reduces the Shannon diversity index of soil bacteria (Figure 1A). Bacterial species have an osmotic adjustment function, but they are more vulnerable to drought because they require water membranes in soil aggregates and on soil surfaces for substrate dispersion and diffusion (Esther and Joseph, 2016). Under drought conditions, the decrease of soil moisture will affect soil porosity, thereby inhibiting the growth and multiplication of bacteria (Farooq et al., 2009). Our paper showed TN substantially affects bacterial diversity (Figure 4). Drought reduces plant productivity and nitrogen fixation capacity (Ma et al., 2020), thereby decreasing the supply of soil TN and limiting the growth and multiplication of bacteria. Under drought stress, soil moisture mobility is poor, affecting soil nutrient mobility (Moyano et al., 2013), and bacteria die because of a lack of sufficient energy sources. In addition, plant diversity considerably affects bacterial diversity (Figure 4), which is consistent with the results of previous studies (Schlatter et al., 2015). Plant diversity induces species-specific effects that may affect bacterial diversity through changes in root exudates, plant litter, and plant secondary metabolites (He et al., 2008).

Microbial responses to drought depend on their metabolic flexibility and physiological conditions. Fungi can remain active at a lower water potential compared with bacteria. The above results also verified our first prediction. Fungi are more resistant to drought than bacteria because they can establish large water absorption networks, which promote long-distance water transfer and enable them to explore water-filled soil pores or obtain water from small soil pores (Sun et al., 2020). In this paper, fungal diversity increased significantly because of drought treatments (Figure 1B), which is consistent with the results of previous studies (de Oliveira et al., 2020). Drought may promote the growth of potentially slow-growing, drought-adapted soil microbes. Changes in fungal community diversity result from fungal redistribution, water use (Barnard et al., 2013), or mycelial contraction (Hossain et al., 2007), leading the community to adopt ecological strategies appropriate to different drought conditions. Our study found that BGB significantly affected fungal diversity (Figure 4), which is consistent with the results of previous studies (Li et al., 2022b). This result may be attributed to two aspects. Firstly, BGB affects belowground nutrient and energy exchange, and the inputted organic and inorganic material by inter–root secretions promotes or inhibits the growth and diversity of soil fungi (Graham and Mendelssohn, 2016). Secondly, mycorrhizal (plant root–fungal symbiosis) mycelium expands the root uptake area to utilize deep soil water (McHugh and Gehring, 2006).

### 4.2. Soil microbial community structure

In this study, dominant bacterial phyla in drought stress treatment were Actinobacteria, Proteobacteria, Chloroflexi, and Acidobacteria, which are common bacterial phyla in soil subjected to drought stress, similar to a meadow steppe (Yang et al., 2021). Drought stress changed the abundance of the microbial dominant phylum (Figure 3), significantly increasing Gram-positive bacteria (i.e., Actinobacteria and Chloroflexi). The relative abundance of the microbial community of Gram-positive bacteria increased in response to drought stress (Fuchslueger et al., 2014). This result may be related to the cell structure of Gram-positive and unique physiological characteristics. Gram-positive bacteria have a thick, tough cell wall outside the cell membrane (Schimel et al., 2007), which is less susceptible to water loss and death under drought stress (Manzoni et al., 2012). On the contrary, the relative abundance of Gram-negative bacteria (i.e., Proteobacteria) were decreased under drought due to loss of sporulation capacities during the course of evolution and poor adaption to soil moisture disturbance (Denef et al., 2009).

Related studies have found that changes in the external environment will influence the function of soil microorganisms and soil microbial community structure until reaching a new nutrient balance condition (Petra et al., 2003). Actinomycetes were significantly and negatively correlated with SWC (Figure 6) and drought tolerance, and they were well enriched in arid environments (Xu et al., 2018). Actinobacteria can adapt to soil environments under prolonged water and nutrient stress because of their ability to decompose soil litter and a variety of organic compounds, including aromatics, cellulose, wood, and other complex compounds (Van Bergeijk et al., 2020) to maintain its growth and reproduction. By contrast, drought reduces the relative abundance of oligotrophic bacteria (i.e., Acidobacteria) as this community is unable to synthesize all important nutrients, promotes decomposition of difficult-to-degrade C sources and acid uptake and grows slowly (Zengler and Zaramela, 2018). Previous studies have shown that Acidobacteria readily multiply in acidic soils (Kim et al., 2021). In the present paper, no significant correlation was observed between Acidobacteria and soil pH probably because the soil pH in the study area was neutral, and drought did not significantly change the pH (Supplementary Table S3). Bacteroidetes are well known degraders of polymeric organic matter, and they are important components of some organic carbon recycling and decomposition (Thomas et al., 2011). Drought reduced the relative abundance of this bacterium group, which is consistent with the results of the Inner Mongolia arid grassland study (Shao et al., 2018). This result may be due the patchiness of grasslands because of drought (Hoffman et al., 2017) and reduced net primary productivity of plants (Shaw et al., 2022). Bacteroidetes lack energy sources to readjust growth strategies of species distribution because of reduced organic carbon input.

Compared with bacteria, fungi have more unique survival skills or physiological structures to increase tolerance (Kaisermann et al., 2015). The effects of drought stress on fungi were mostly concentrated in Basidiomycota and Ascomycota. In this study, drought reduced the relative abundance of Basidiomycota (*p* < 0.05, Figure 3). Some of the Basidiomycota colonies form symbiotic associations with root systems of specific plants (Lareen et al., 2016), and reduced water input may weaken the cooperative relationship. The results of this study showed that SWC was significantly positively correlated with Basidiomycota (Figure 6), confirming the existence of ectomycorrhizal mycorrhizal cooperation between Basidiomycota and plant roots. Drought reduced water transport and nutrients by fungal mycelium for plants outside the root system, and the regulatory and storage role of plant roots is reduced (Naylor and Coleman, 2018), thereby affecting Basidiomycota growth. However, drought increased the relative abundance Ascomycota (*p* < 0.05, Figure 3). Thus, ascomycete fungi might produce ascospores adapted to the drought environment (Lozano et al., 2021).

Drought stress affects the community structure of soil microorganisms (bacteria and fungi) by affecting various environmental factors such as soil organic carbon, organic nitrogen, soil aeration, and pH value (Yang et al., 2021). In this study, the RDA correlation analysis in Figure 5 shows that SWC is the main soil factor affecting the soil microbial community structure (Figures 5A,C). Water primarily affects the growth and vitality of plant roots and changes the content of root exudates (Fan et al., 2019). It also affects the bacterial and fungal diversity in soil. Moreover, the plant diversity (Shannon Wiener and Pieiou) and AGB were significantly associated with the bacterial and fungal community structure (Figures 5B,D). Plant diversity enriches the soil microbial community structure, and microbial community affects plant growth by changing nutrient supply (Bijalwan et al., 2022). When plant community diversity is poor, the composition of litter and root exudates decreases, and the structure of soil microbial community changes (Mendes et al., 2013). The above results also verified our second prediction. Consequently, the change in precipitation gradient (drought stress) plays an important role in the construction of soil microbial diversity in the alpine grassland region.

### 4.3. Soil microbial predicted functional potential

Drought stress changed the nutrient balance of the soil microbial community structure, inevitably causing the soil microbial community function shift until maintaining a certain nutrient balance (Petra et al., 2003). For the bacterial predicted functional potential, drought stress showed substantially influence on three level-1 functional categories (i.e., Metabolism, Genetic information processing, and Cellular processes), and nine level-2 functional categories were significantly different under CK, D20, and D40 (Supplementary Table S5). The reason maybe that due to the weak resistance of bacteria to drought (Zhang et al., 2016; Kaisermann et al., 2017), the diversity decreased, and the community structure composition began to change under D20 treatment (Figures 1A, 3), which also can be confirmed by results of the co-occurrence networks of bacterial taxa at the genus level (Supplementary Figures S7–S9; Supplementary Table S6). The numbers of nodes, total links, positive links, network centralization, and network density in the bacterial networks are decreased with drought degree (Supplementary Table S6), indicating that the stability and interaction of bacterial communities were severely impaired by drought stress. Research showed the microbial functional potential is largely determined by microbial community composition (Naidoo et al., 2022). Furthermore, under excessive drought treatment (D40), more dead bacterial residues were transformed into SOM and nutrients, which can be utilized by saprophytic fungi (Sokol et al., 2022). Thus, compared with CK and D20, D40 showed a higher correlation with SOM, Species Richness, and Shannon Wiener (Figure 8A). Drought stress changed SWC and plant diversity in the plots, altered the bacterial community composition and further indirectly affected the cell movement, metabolism, and genetic information processing in the bacterial community.

For the fungal predicted functional potential, with the aggravation of drought, the proportion of pathogen-saprotroph, parasite–saprotroph, and endophyte–saprotroph fungi functions increased (Supplementary Figure S6; Table 1), indicating that the resistance of fungi to drought was disintegrated. The function shift in fungal community was also mainly due to the changes of community composition (Naidoo et al., 2022). Excessive drought led to a substantial increase in the proportion of Ascomycete and Glomeromycota fungi that mainly engaged in saprophytic, parasitic, and symbiotic modes (Figure 3). Consistent with previous studies (Zhang et al., 2016; Kaisermann et al., 2017; Franciska et al., 2018), fungi were more drought-resistant than bacteria (Figures 1B, 2B). Drought stress leads to the death of plants and bacteria, promoted the enrichment of SOM and enhanced the saprotroph function under D20 (Franciska et al., 2018; Sokol et al., 2022). However, excessive drought (D40) led to the rapid death of some fungi (i.e., Basidiomycota), changed the fungal community composition (Figure 3) and then altered the function of fungi shifting from saprotroph to pathogen–saprotroph and parasite-saprotroph symbiosis (Sokol et al., 2022). The highest positive links and smallest shortest paths in D40 of fungal networks also confirmed synergistic interaction of multiple fungal genera (Supplementary Figures S10–S12; Supplementary Table S6). Thus, the functions of fungal communities under excessive drought stress showed remarkable dependence on SOM and Shannon Wiener (Figure 8B). Drought stress changed SWC and plant diversity in the plots, altered the fungal community composition, and further indirectly affected the function shift (saprotroph, pathogen–saprotroph, endophyte–saprotroph, and parasite–saprotroph) in the fungal community. The above results also verified our third prediction. Consistent with previous findings, taxonomy and function were coupled (Fierer et al., 2012). Although the above functions shift may not necessarily simply relate to microbial community composition (such as inevitable adaptive gene loss, convergent evolution, and horizontal gene transfer; Louca et al., 2018), these results in our study indicate that the soil microbial functional potential could be predictable through taxonomic community profiles.

## 5. Conclusion

In this study, the effects of drought stress on soil microbial diversity, community composition, and predicted functional potential in alpine grasslands of Kunlun Mountains were investigated and determined. Our results showed bacteria and fungi responded differently to drought intensity, and bacteria were more sensitive to drought compared with fungi. Therefore, the diversity or structure of soil bacteria community could serve as an indicator of alpine grasslands status, with practical significance for alpine grassland ecosystem development. However, the fungal community still had difficulty maintaining resistance under excessive drought stress. Notably, soil moisture content, plant diversity (Shannon Wiener, Pieiou, and Simpson), and SOM were the main drivers affecting soil microbial community structure composition and functional potential, which provided a new perspective for the management of alpine grasslands. This work also confirmed that the soil microbial predicted functional potential could be predictable through taxonomic community profiles. Our findings improved the comprehensive understanding about the different responses of soil microbial diversity, community composition, and functional potential to drought stress in a semiarid alpine grassland and provide a theoretical basis for exploring the mechanism of microbial response to climate change in alpine grassland ecosystems.

## Data availability statement

The datasets presented in this study can be found in online repositories. The names of the repository/repositories and accession number(s) can be found at: https://www.ncbi.nlm.nih.gov/, PRJNA881479.

## Author contributions

QW analyzed data and wrote the manuscript. ZZ and YL carried out the experiments and generated the data. LB and MX analyzed the data. LL conceived the work, designed the experiment, and supervised this research. All authors contributed to the article and approved the submitted version.

## Funding

This research was supported by the Youth Innovation Promotion Association of the Chinese Academy of Sciences (2020434), Shandong Provincial Natural Science Foundation (ZR2020MC040), Shandong Province College Youth Innovation Technology Support Program (2020KJE009), National Natural Science Foundation of China (41807335), and National Postdoctoral Program for Innovative Talents (BX201700279).

## Conflict of interest

The authors declare that the research was conducted in the absence of any commercial or financial relationships that could be construed as a potential conflict of interest.

## Publisher’s note

All claims expressed in this article are solely those of the authors and do not necessarily represent those of their affiliated organizations, or those of the publisher, the editors and the reviewers. Any product that may be evaluated in this article, or claim that may be made by its manufacturer, is not guaranteed or endorsed by the publisher.

## Footnotes

^1^http://www.ncbi.nlm. nih.gov/PRJNA881479

^2^ http://scikit-bio.org

^3^ https://www.genescloud.cn

^4^ https://cytoscape.org/
