# Supplementary material for: Different and unified responses of soil bacterial and fungal community composition and predicted functional potential to 3 years’ drought stress in a semiarid alpine grassland
Source: Front Microbiol. 2023 Mar 14;14:1104944. doi: 10.3389/fmicb.2023.1104944 (PMC10112540; doi:10.3389/fmicb.2023.1104944)
Supplement: Supplementary file 1 [file Data_Sheet_1.docx]

Supplementary Material

Different and unified responses of soil bacterial and fungal community composition and predicted functional potential to three years’ drought stress in a semi-arid alpine grassland

Qian Wan, Lei Li*, Bo Liu, Zhihao Zhang, Yalan Liu, Mingyu Xie

*** Corresponding authors:**

Dr. Lei Li, State Key Laboratory of Desert and Oasis Ecology, Xinjiang Institute of Ecology and Geography, Chinese Academy of Sciences, Urumqi 830011, China. Tel: +86 18690230972, E-mail: [lilei@ms.xjb.ac.cn](mailto:lilei@ms.xjb.ac.cn)

**Table S1.** Precipitation statistics for the northern slopes of the Kunlun Mountains from March to October 2019-2021.

| Month | 2019 (mm) | 2020 (mm) | 2021 (mm) |
| --- | --- | --- | --- |
| March | 0.1 | 1.1 | 4.5 |
| April | 8.8 | 65.7 | 24.8 |
| May | 40 | 95.9 | 56.2 |
| June | 163.8 | 101.1 | 81.4 |
| July | 82.2 | 32.1 | 44.3 |
| August | 74.4 | 7.6 | 39.7 |
| September | 40.9 | 1.9 | 12.1 |
| October | 15 | 1.5 | 11.2 |
| Total | 425.2 | 306.9 | 274.2 |


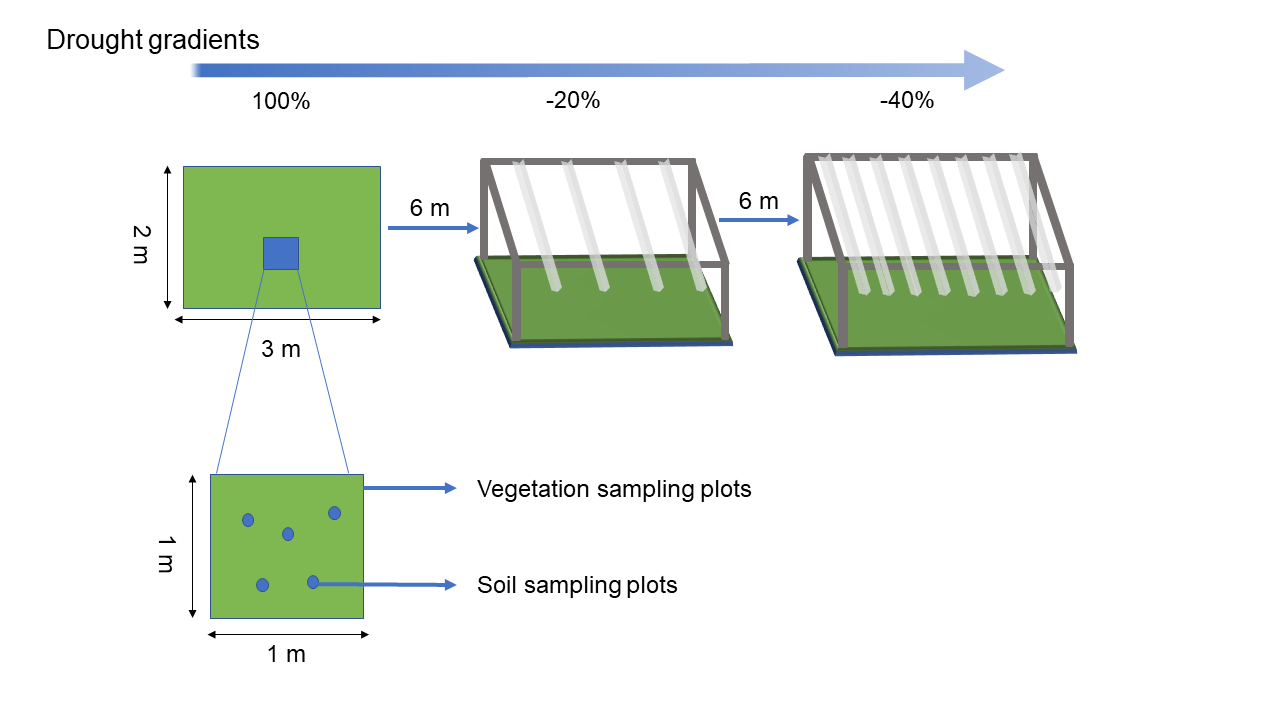


**Fig S1.** Experimental design for vegetation and soil sampling. 100% = without throughfall reduction, -20 = 20% throughfall reduction, and -D40 = 40% throughfall reduction.


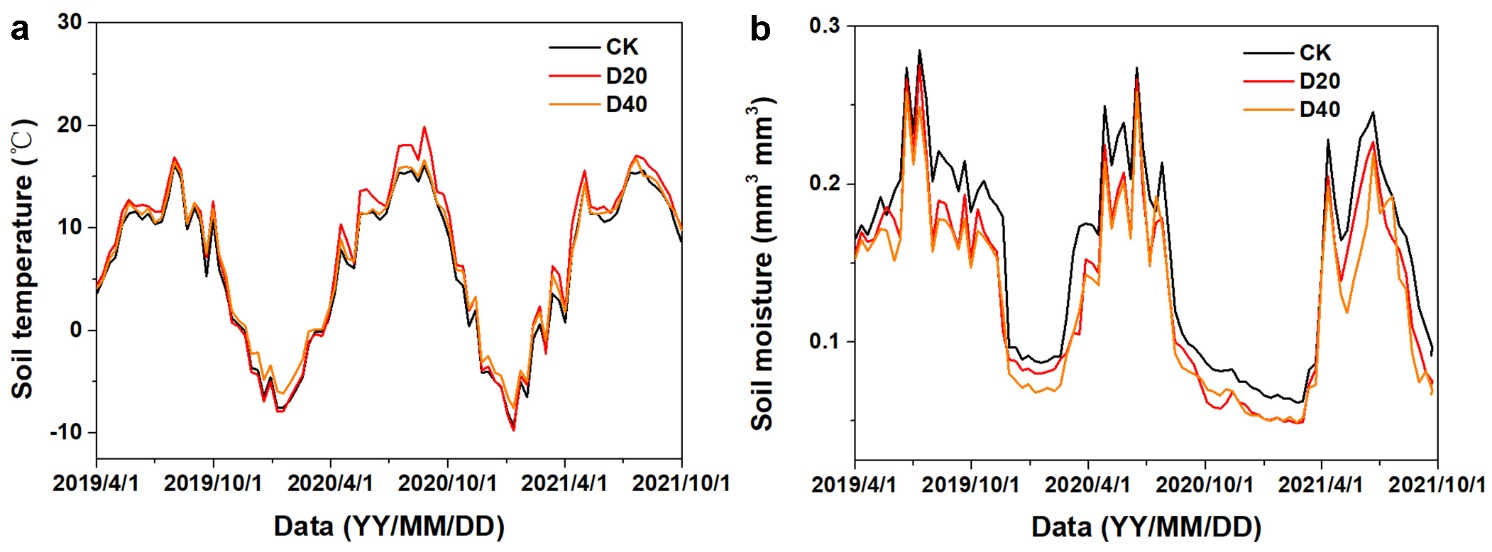


**Fig S2.** Soil temperature (a) and soil moisture (b) at a 15 cm soil depth for the control and drought treatments from 2019 to 2021. CK = without throughfall reduction, D20 = 20% throughfall reduction, and D40 = 40% throughfall reduction.

**Table S2.** The plant community density (plants/m^2^) characteristics of the northern slopes in Kunlun Mountains under different stages of precipitation. Values are means ± SE (n = 4).

| Family | vegetation types | CK | D20 | D40 |
| --- | --- | --- | --- | --- |
| Compositae | *Seriphidium rhodanthum* | 39.12±13.4 | 42.31±9.3 | 41.16±7.7 |
| Gramineae | *Stipa capillata* | 39.28±11.7 | 25.19±5.3 | 35.27±9.3 |
|  | *Festuca ovina* L. | 10.41±2.6 | 13.14±3.0 | 12.09±2.8 |
| Liliaceae | *Allium chrysanthum* R. | 7.44±5.7 | 9.51±5.3 | 5.75±3.8 |
| Leguminosae | *Astragalu spolycladus* | 2.13±1.1 | 2.71±1.2 | 2.16±1.2 |

**Table S3.** Changes in soil properties and the vegetation survey in different stages of precipitation. Values are means ± SE (n = 4). SOM, soil organic matter; SAN, soil alkali-hydrolysable nitrogen; SAP, available phosphorous; TK, total soil potassium; TC, total soil carbon; TN, total soil nitrogen; TP, total soil phosphorous; soil total C: total N, soil total C: total P and soil total N: total P; SWC, soil moisture content; ST, soil temperature; AGB, above ground biomass; BGB, below ground biomass. Different lowercase letters (a, b, ab, c) indicate significant differences among different treatments (ANOVA *P* < 0.05).

|  | CK | D20 | D40 |
| --- | --- | --- | --- |
| SOM (g kg^-1^) | 17.42±2.62 | 16.54±2.62 | 19.52±2.91 |
| pH | 7.58±0.01 | 7.62±0.03 | 7.65±0.02 |
| SAN (mg kg^-1^) | 36.84±1.91 | 34.89±0.99 | 35.48±0.53 |
| SAP (mg kg^-1^) | 1.08±1.16 | 1.16±0.50 | 1.13±0.19 |
| TK (g kg^-1^) | 5.95±0.11 | 6.12±0.02 | 5.99±0.08 |
| TC | 10.10±1.41 | 9.59±1.04 | 11.32±1.76 |
| TN (g kg^-1^) | 0.75±0.07 | 0.70±0.03 | 0.66±0.08 |
| TP (g kg^-1^) | 0.63±0.33 | 0.61±0.02 | 0.65±0.03 |
| C:N | 14.93±0.82 | 14.61±1.17 | 16.33±1.65 |
| N:P | 1.12±0.60 | 1.01±0.62 | 1.13±0.41 |
| C:P | 16.11±0.93^a^ | 14.70±0.97^b^ | 18.37±1.26^ab^ |
| ST (℃) | 11.83±0.01 | 11.14±0.37 | 10.98±0.28 |
| SWC (%) | 21.02±0.73^a^ | 18.58±0.47^b^ | 16.32±0.35^c^ |
| AGB (g m^-2^) | 176.41 ±12.22 | 118.51±7.02 | 150.79±8.04 |
| BGB (g m^-2^) | 43.97±2.55 | 35.04±9.31 | 57.23±14.58 |
| Species Richness | 4.50±0.02 | 4.75±0.50 | 4.75±0.40 |
| Shannon Wiener | 1.57±0.04 | 1.61±0.03 | 1.49±0.02 |
| Pieiou (H) | 0.87±0.03 | 0.90±0.01 | 0.83±0.04 |
| Simpson (D) | 0.72±0.01 | 0.75±0.01 | 0.76±0.02 |
| Coverage (%) | 53±8.04 | 60±4.87 | 53±7.12 |

**Table S4.** Primer sets and thermal profiles used in PCR amplification.

| Target group | Primer | Sequence (5′­­−3′) | PCR conditions |
| --- | --- | --- | --- |
| Bacterial 16S_V3V4 | 338F | ACTCCTACGGGAGGCAGCA | 2 min at 95 °C followed by 30 cycles of 30 s at 95 °C, 45 s at 55 °C, 60 s at 72 °C, and 10 min at 72 °C for the last cycle. |
|  | 806R | GGACTACHVGGGTWTCTAAT |  |
| Fungal  ITS_V1 | ITS5 | GGAAGTAAAAGTCGTAACAAGG | 3 min at 95 °C followed by 35 cycles of 30 s at 95 °C, 35 s at 56 °C, 50 s at 72 °C, and 10 min at 72 °C for the last cycle (Li et al., 2014). |
|  | ITS2 | GCTGCGTTCTTCATCGATGC |  |


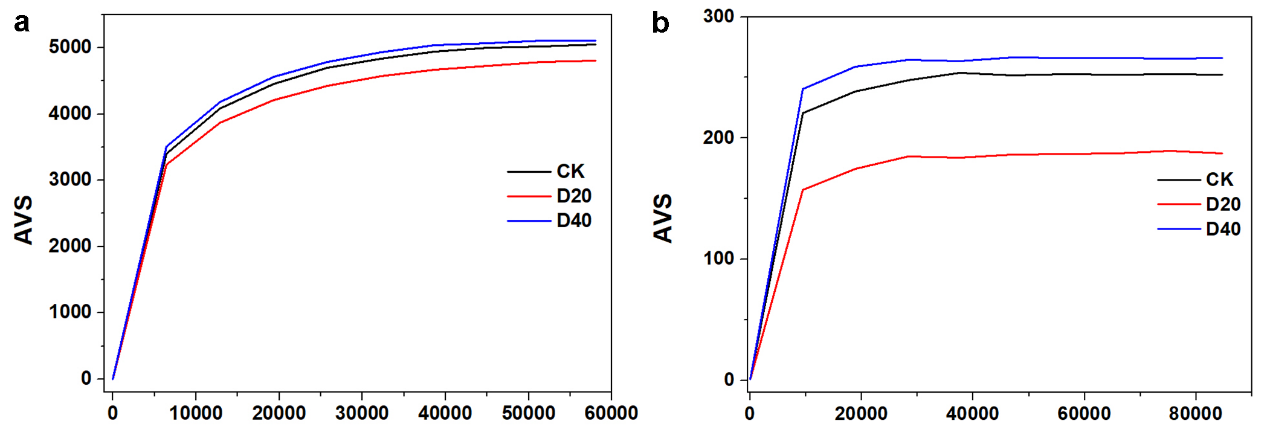


**Fig S3.** Rarefaction curve of soil bacteria (a) and fungus (b).





**Fig** **S4.** Effect of different drought gradients on the species richness of soil bacteria and fungi. Different letters (a, b) indicate significant differences based on *P* < 0.05.


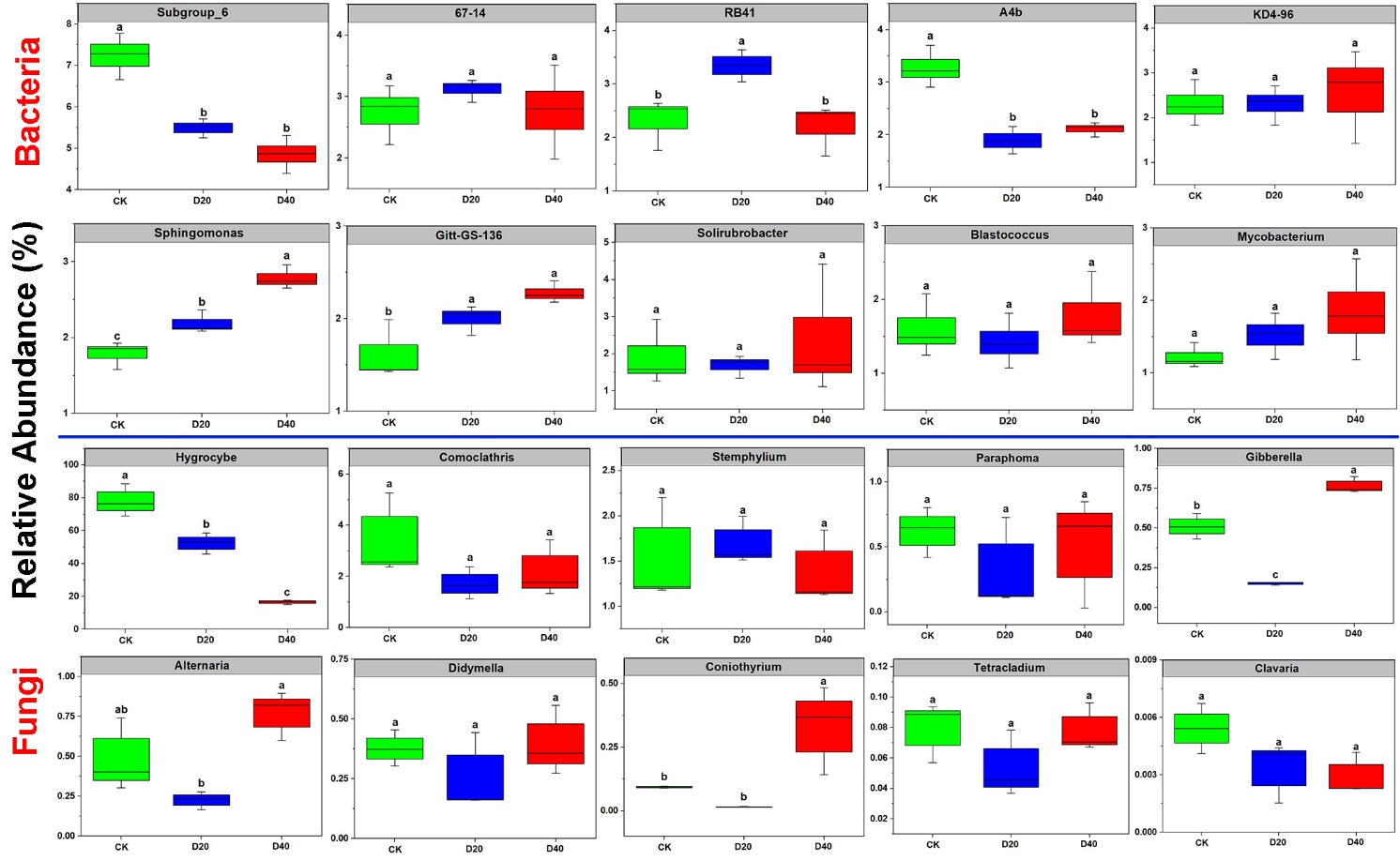


**Fig S5.** The relative abundance of soil bacterial/fungal communities at the genus levels under different drought treatments. Different letters (a, b, ab, c) indicate significant differences based on *P* < 0.05.

**Table S5.** Changes of predicted soil bacterial functional groups in different treatments. Different letters (a, b, ab) indicate significant differences among different treatments (*P* < 0.05). Values are means ± SD (n = 4).

| Bacterial functional groups | CK (%) | D20 (%) | D40 (%) |
| --- | --- | --- | --- |
| Cell growth and death | 1.27±0.01b | 1.30±0.01a | 1.30±0.01a |
| Cell motility | 1.89±0.06a | 1.83±0.02ab | 1.78±0.04b |
| Transport and catabolism | 0.39±0.01a | 0.37±0.01b | 0.39±0.01a |
| Replication and repair | 4.94±0.03a | 4.83±0.04b | 4.87±0.01b |
| Translation | 2.60±0.01a | 2.56±0.01b | 2.58±0.01ab |
| Amino acid metabolism | 13.33±0.01b | 13.38±0.01a | 13.40±0.03a |
| Biosynthesis of other secondary metabolites | 2.86±0.01a | 2.82±0.01b | 2.86±0.01a |
| Carbohydrate metabolism | 13.58±0.01b | 13.56±0.01b | 13.69±0.03a |
| Lipid metabolism | 7.60±0.07a | 7.59±0.02a | 7.40±0.02b |

**
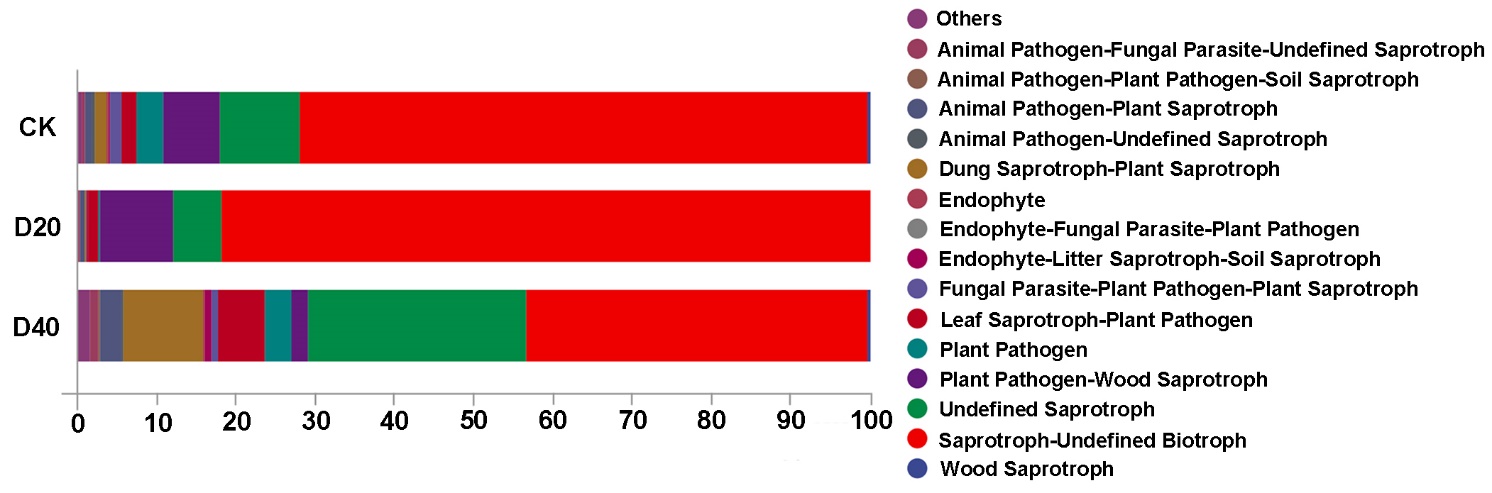
Fig S6.** Relative abundance of functional categories for different treatments in three origins fungal microbiome using FUNGuild.

**
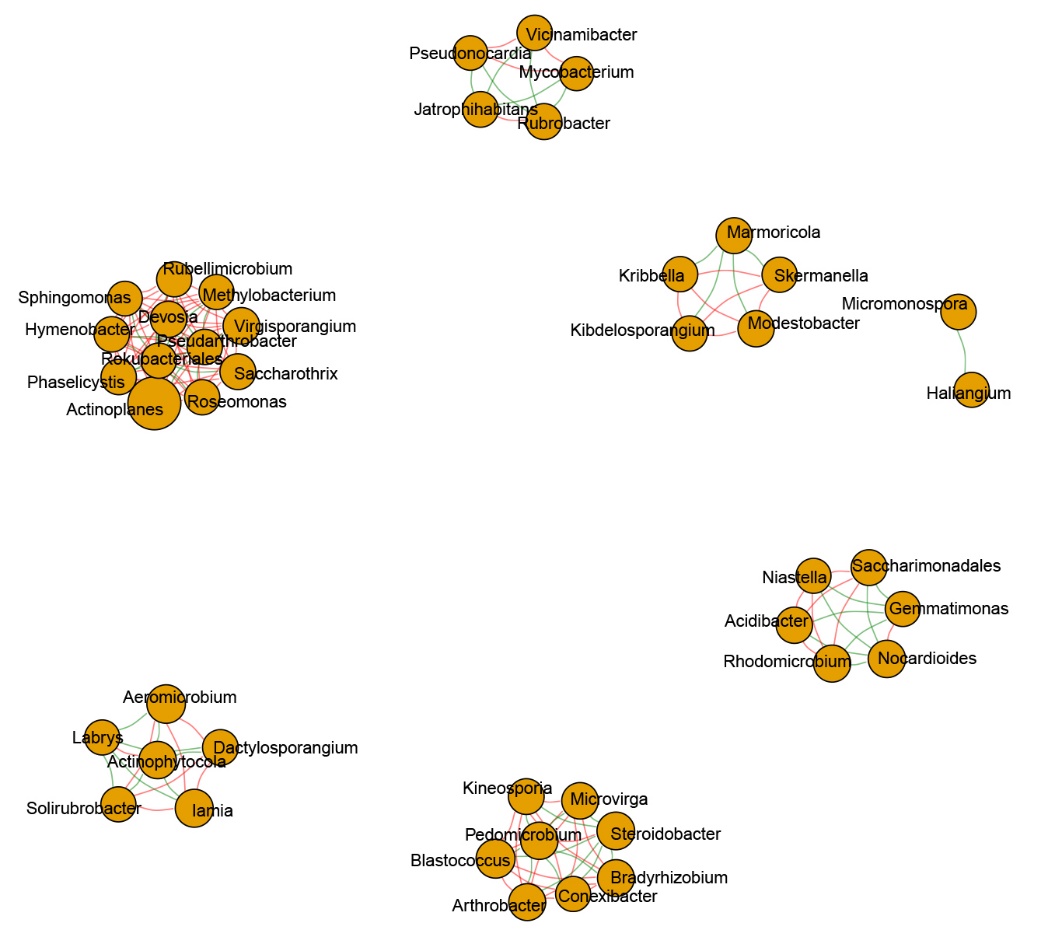
**

**Fig S7.** Co-occurrence networks of bacterial taxa in CK based on operational taxonomic units (OTUs) at the genus level according to the Spearman correlation coefficients.

**
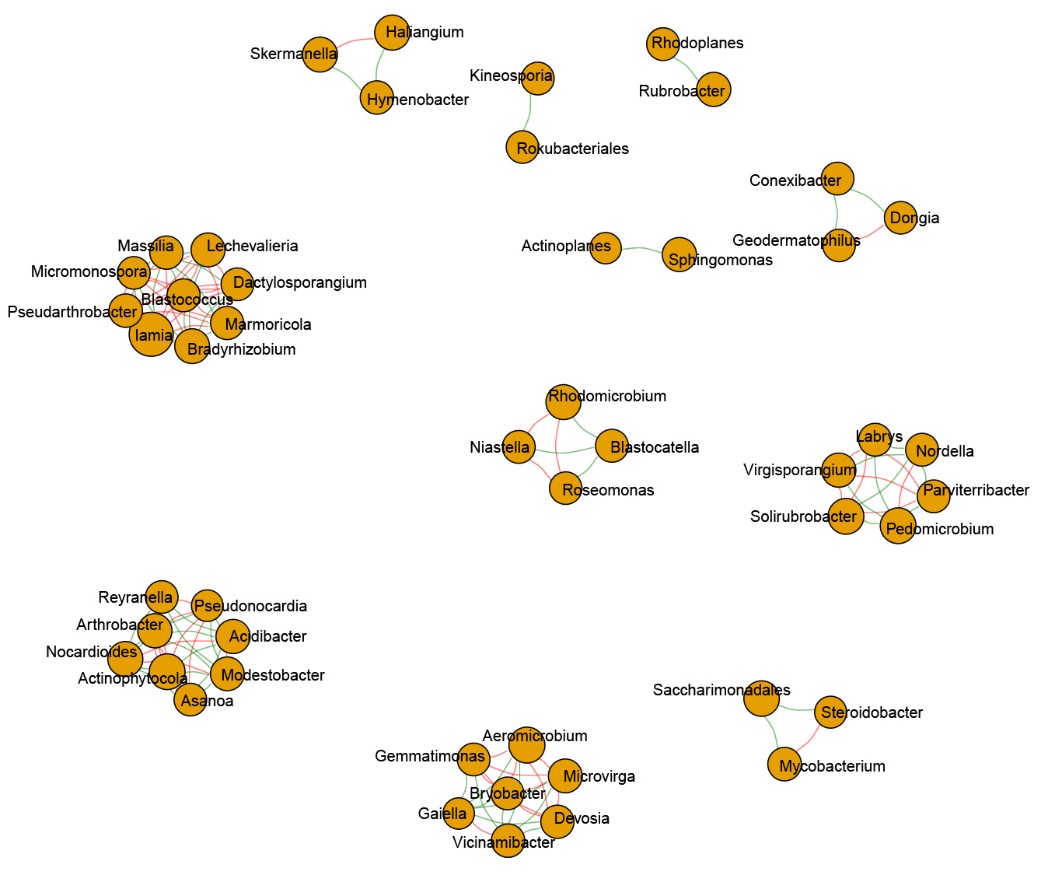
**

**Fig S8.** Co-occurrence networks of bacterial taxa in D20 based on operational taxonomic units (OTUs) at the genus level according to the Spearman correlation coefficients.

**
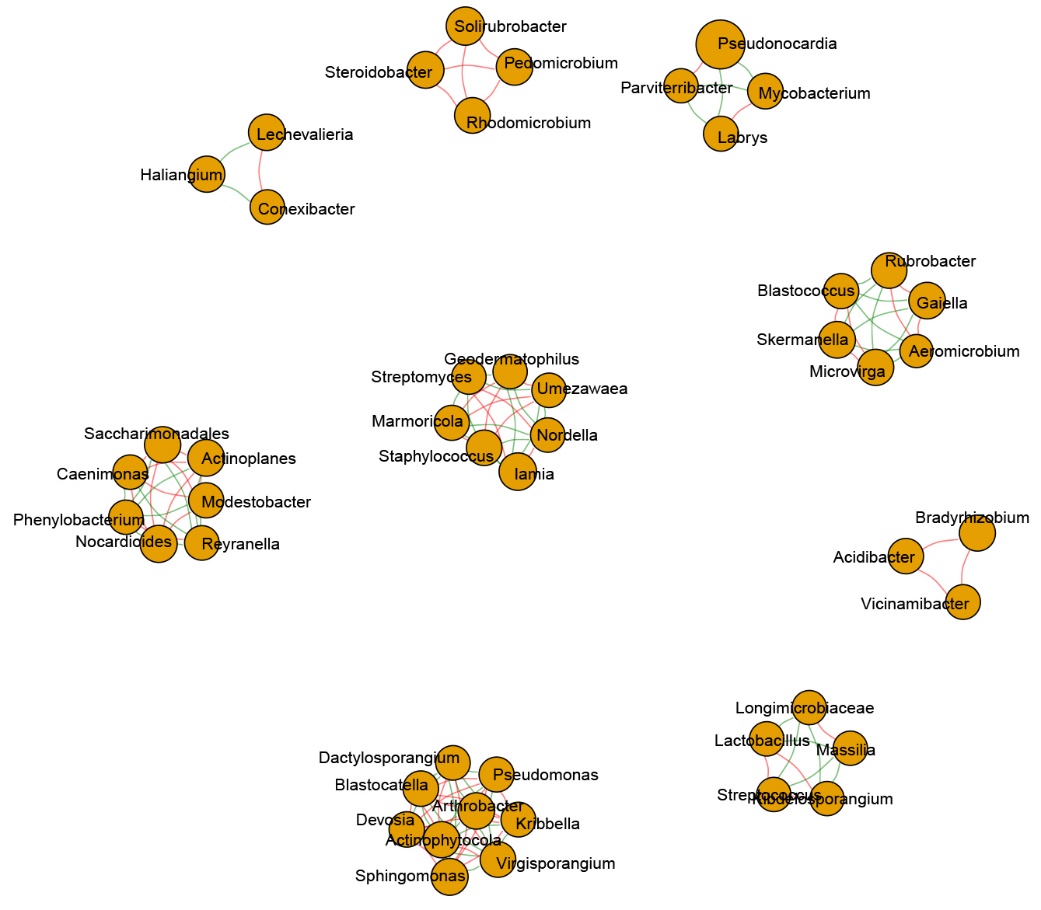
**

**Fig S9.** Co-occurrence networks of bacterial taxa in D40 based on operational taxonomic units (OTUs) at the genus level according to the Spearman correlation coefficients.

**
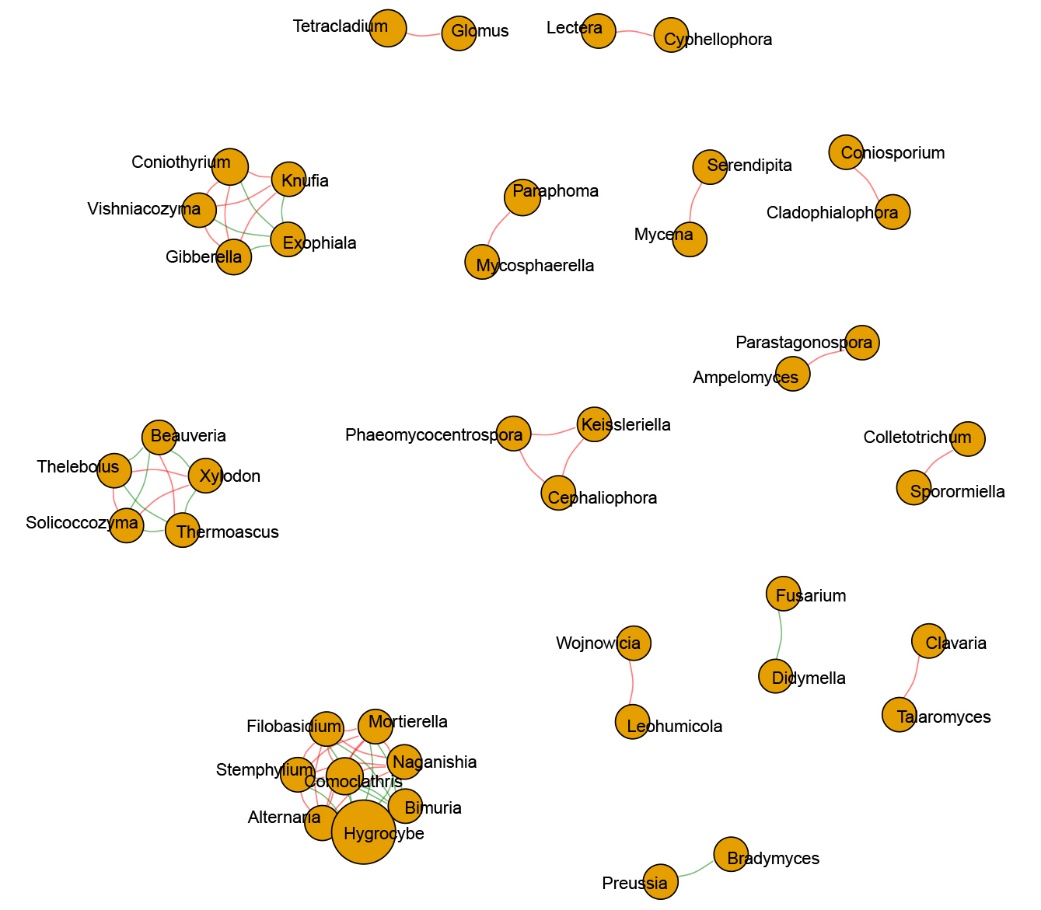
**

**Fig S10.** Co-occurrence networks of fungal taxa in CK based on operational taxonomic units (OTUs) at the genus level according to the Spearman correlation coefficients.

**
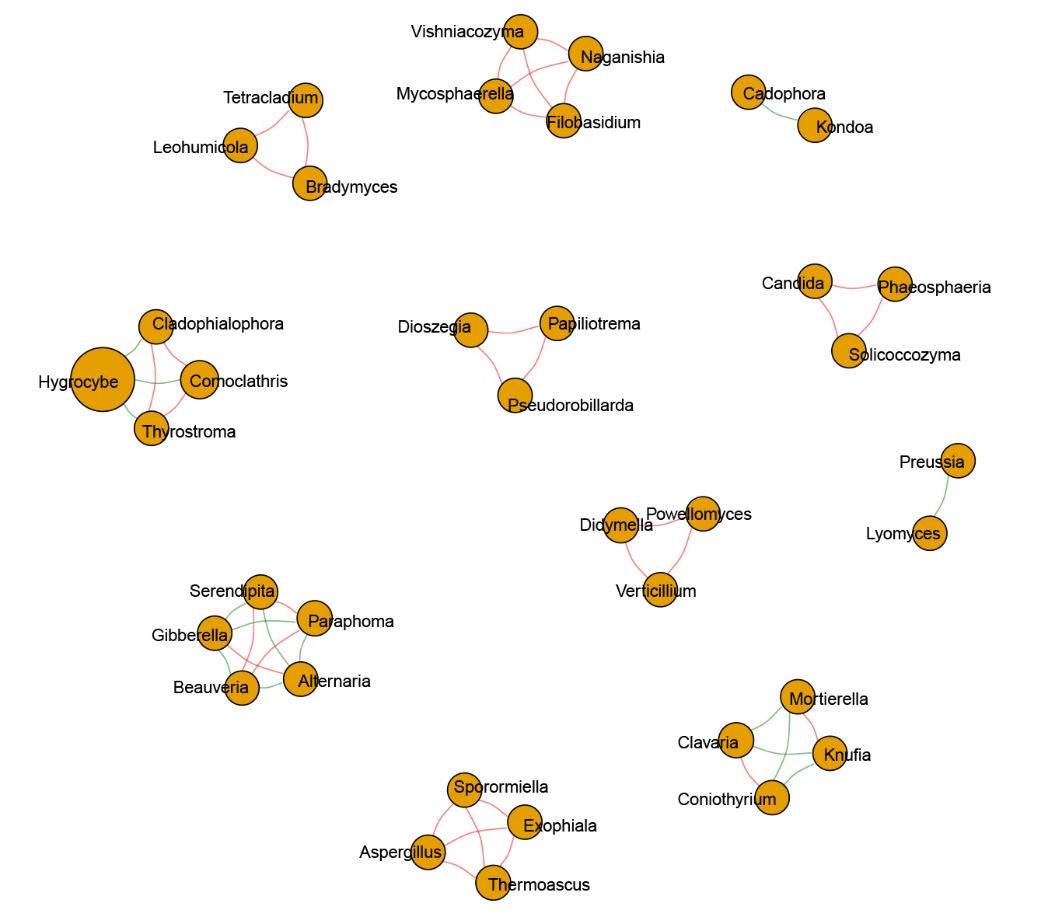
**

**Fig S11.** Co-occurrence networks of fungal taxa in D20 based on operational taxonomic units (OTUs) at the genus level according to the Spearman correlation coefficients.

**
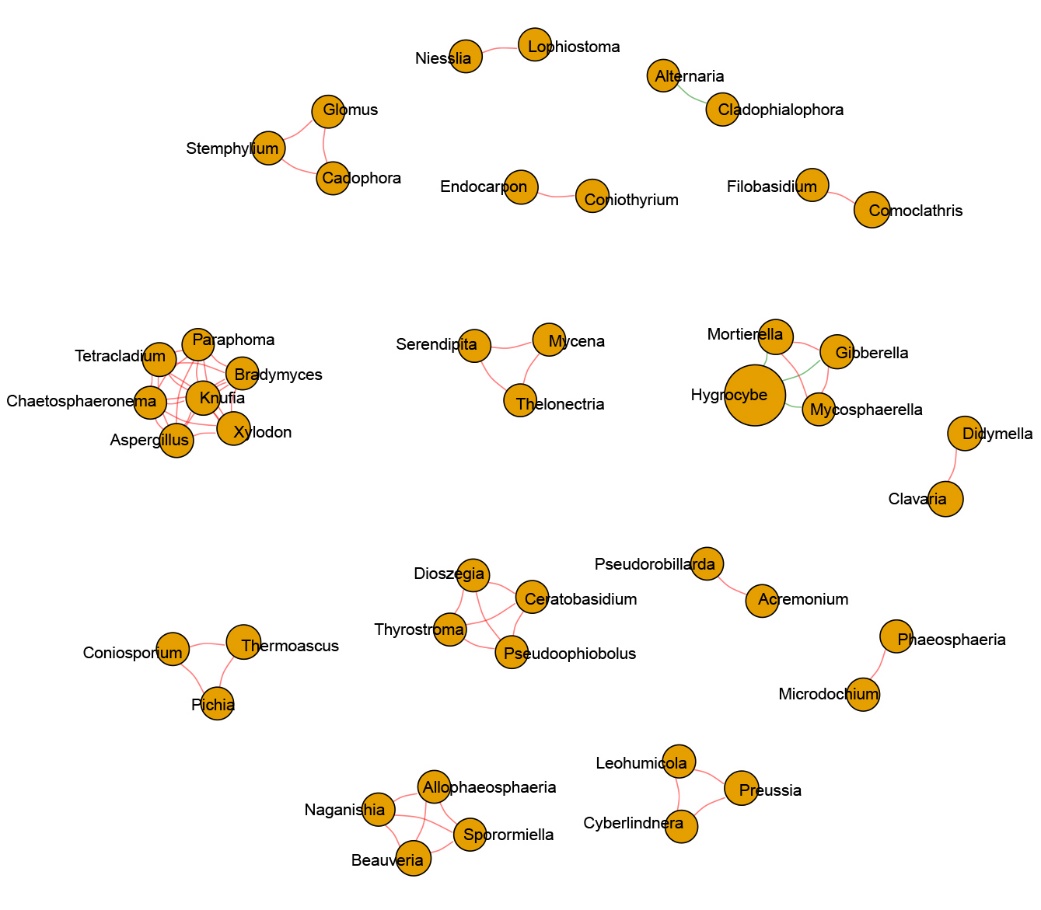
**

**Fig S12.** Co-occurrence networks of fungal taxa in D40 based on operational taxonomic units (OTUs) at the genus level according to the Spearman correlation coefficients.

**Table S6.** Soil bacterial and fungal network properties in different treatments.

| Network  Parameters | Bacterial | | | Fungal | | |
| --- | --- | --- | --- | --- | --- | --- |
|  | CK | D20 | D40 | CK | D20 | D40 |
| Nodes | 44 | 48 | 48 | 43 | 37 | 45 |
| Links | 145 | 121 | 118 | 62 | 48 | 58 |
| Positive Links | 91 | 66 | 57 | 38 | 33 | 54 |
| Negative Links | 54 | 57 | 61 | 24 | 15 | 4 |
| Network centralization | 0.107 | 0.069 | 0.066 | 0.103 | 0.041 | 0.081 |
| Network density | 0.153 | 0.107 | 0.100 | 0.069 | 0.072 | 0.059 |
| Network heterogeneity | 0.454 | 0.523 | 0.390 | 0.799 | 0.329 | 0.641 |
| Shortest paths | 290(15%) | 242(10%) | 236(10%) | 124(6%) | 96(7%) | 116(5%) |
| Average number of neighbors | 6.591 | 4.816 | 5.042 | 2.884 | 2.595 | 2.578 |
| Clustering coefficient | 0.955 | 0.878 | 1.0 | 0.488 | 0.892 | 0.689 |

# References

Li, X., Rui, J., Mao, Y., Yannarell, A., and Mackie, R. (2014). Dynamics of the bacterial community structure in the rhizosphere of a maize cultivar. *Soil Biol. Biochem.* 68, 392-401.
